# Effects of a brief video intervention on treatment initiation and adherence among patients attending human immunodeficiency virus treatment clinics

**DOI:** 10.1371/journal.pone.0204599

**Published:** 2018-10-05

**Authors:** Mary Spink Neumann, Aaron Plant, Andrew D. Margolis, Craig B. Borkowf, C. Kevin Malotte, Cornelis A. Rietmeijer, Stephen A. Flores, Lydia O’Donnell, Susan Robilotto, Athi Myint-U, Jorge A. Montoya, Marjan Javanbakht, Jeffrey D. Klausner

**Affiliations:** 1 Centers for Disease Control and Prevention, National Center for HIV/AIDS, Viral Hepatitis, STD, and TB Prevention, Division of HIV/AIDS Prevention, Atlanta, GA, United States of America; 2 Sentient Research, West Covina, CA, United States of America; 3 California State University-Long Beach, Long Beach, CA, United States of America; 4 Colorado School of Public Health, University of Colorado, Denver, CO, United States of America; 5 Education Development Center, Waltham, MA, United States of America; 6 Health Resources and Services Administration, HIV/AIDS Bureau, Rockville, MD, United States of America; 7 University of California-Los Angeles, Los Angeles, CA, United States of America; Asociacion Civil Impacta Salud y Educacion, PERU

## Abstract

**Background:**

Persons with human immunodeficiency virus (HIV) who get and keep a suppressed viral load are unlikely to transmit HIV. Simple, practical interventions to help achieve HIV viral suppression that are easy and inexpensive to administer in clinical settings are needed. We evaluated whether a brief video containing HIV-related health messages targeted to all patients in the waiting room improved treatment initiation, medication adherence, and retention in care.

**Methods and findings:**

In a quasi-experimental trial all patients (N = 2,023) attending two HIV clinics from June 2016 to March 2017 were exposed to a theory-based, 29-minute video depicting persons overcoming barriers to starting treatment, taking medication as prescribed, and keeping medical appointments. New prescriptions at index visit, HIV viral load test results, and dates of return visits were collected through review of medical records for all patients during the 10 months that the video was shown. Those data were compared with the same variables collected for all patients (N = 1,979) visiting the clinics during the prior 10 months (August 2015 to May 2016). Among patients exposed to the video, there was an overall 10.4 percentage point increase in patients prescribed treatment (60.3% to 70.7%, p< 0.01). Additionally, there was an overall 6.0 percentage point improvement in viral suppression (56.7% to 62.7%, p< 0.01), however mixed results between sites was observed. There was not a significant change in rates of return visits (77.5% to 78.8%). A study limitation is that, due to the lack of randomization, the findings may be subject to bias and secular trends.

**Conclusions:**

Showing a brief treatment-focused video in HIV clinic waiting rooms can be effective at improving treatment initiation and may help patients achieve viral suppression. This feasible, low resource-reliant video intervention may be appropriate for adoption by other clinics treating persons with HIV.

**Trial registration:**

http://www.ClinicalTrials.gov (NCT03508310).

## Introduction

Early initiation of antiretroviral therapy (ART) for patients infected with the human immunodeficiency virus (HIV) and subsequent lifelong viral suppression have important clinical and prevention benefits. [1, 2] HIV treatment and prevention guidelines stress the importance of HIV testing, linking persons diagnosed with HIV infection to care programs, and initiation and maintenance of ART. [3–5] Despite these guidelines, a substantial number of persons with HIV (PWH) in the United States have not been engaged in HIV care services. Centers for Disease Control and Prevention (CDC) data from 2014 show that of the estimated 1.1 million PWH, 51% were not virally suppressed, [6] including an estimated 57% of black, 52% of Hispanic, and 43% of white PWH. Unsuppressed HIV infection, whether from lack of diagnosis, treatment, or medication adherence, is the most important factor contributing to HIV incidence, currently estimated at >35,000 new infections per year in the United States. [6] Although treatment initiation, medication adherence, and retention in care are key for controlling HIV infection from clinical and prevention perspectives, there are few evidence-based behavioral interventions to support these behaviors, especially among black and Hispanic PWH.

CDC’s *Compendium of Evidence-Based Interventions and Best Practices for HIV Prevention* has identified 24 interventions with varying levels of efficacy for improving either medication adherence (N = 14) or retention in, linkage to, and re-engagement in HIV care (N = 11) among PWH. [7] Whether delivered to individuals or groups, these interventions all require multi-session attendance and relatively intensive organizational resources to implement. There is a clear need for simple adherence and retention interventions that can be sustained at relatively low cost and brought to scale quickly to achieve maximum patient coverage. [8]

Two widely disseminated video-based interventions, *VOICES/VOCES* (*V/V*) and *Safe in the City* (*SITC*), were tested in sexually transmitted disease (STD) clinics and found to be cost-beneficial and effective in reducing STD incidence. [9–14] It is therefore reasonable to expect that a waiting room video having a similar format and user-informed development process as *V/V* and *SITC* [15] would have a positive effect on HIV-related health outcomes and be acceptable to both patients and clinic staff. The 25-minute *V/V* [11] and *SITC* [16] education-entertainment videos are available in English and Spanish, can be viewed in clinic waiting rooms or small group sessions, and comprise a set of intervention videos. Drawing on the successful model developed for this set of videos, we created *Taking Care of Me* (*TCOM*), an intervention video with supporting posters for use in HIV clinic waiting rooms. This paper describes its impact on treatment initiation, medication adherence, and retention in care.

## Methods

We used a commercial institutional review board (IRB) because CDC was not involved in data collection and none of the study sites had an affiliated IRB. On April 6, 2016 the IRB approved the study with a waiver of informed consent since patients and providers would not be actively recruited or surveyed, and only routinely collected data from each clinic’s EMR would be used (S1 Appendix). The intervention was registered as a clinical trial after data collection began because no participants were enrolled in the study. The authors confirm that all ongoing and related trials for this intervention are registered.

IRB information:

Western Institutional Review Board | 1019 39th Avenue SE Suite 120 | Puyallup, WA 98374–2115 Office: (360) 252–2500 | Toll Free: (800) 562–4789 www.wirb.com

Study number: 1163249

Protocol number: 20160574

Online tracking: 11–434725

Trial registration: http://www.ClinicalTrials.gov (NCT03508310)

### Patients and sites

To be eligible for participation, HIV clinics had to be located in a community with high AIDS prevalence, use an electronic medical record (EMR) system, have a patient population that is >55% black or African American (hereafter referred to as black) and/or Hispanic or Latino, and agree not to participate in another behavioral intervention study during our study. Also, the clinic’s population had to include >500 HIV-infected patients. Using these criteria, we selected one HIV clinic in Huntsville, AL, another in Atlanta, GA and an HIV case management center affiliated with an HIV clinic in Miami, FL. All patients visiting the clinics or center during the 20-month study period were included in the study.

### Intervention

The *TCOM* video follows the *V/V* and *SITC* model of incorporating key prevention messages into dramatic soap-opera style content involving diverse characters. [16] Multiple script iterations were reviewed by agency administrators, front-line providers, and consultants who were PWH. *TCOM*’s conceptual framework incorporates Social Cognitive Theory, Information-Motivation-Behavioral Skills model, and Social Action Theory, which together address cognitive and behavioral factors related to study outcomes. [10, 11, 13] Storylines embedded prevention messages aimed at increasing treatment initiation (n = 9), medication adherence (n = 35), retention in care (n = 22), partner protection (n = 12), and communication with health care providers (n = 7). The final script presented the prevention messages in dialog or visuals (e.g., taking medication with water instead of alcohol). The resulting 29-minute *TCOM* video includes three vignettes and a 2-part animation about main characters who model overcoming challenges to optimal HIV care. The video was played on continuous loop in recognition of typically short patient wait times. Waiting room posters used images from *TCOM* to direct patients’ attention to the video and reinforce prevention messages.

### Study design

*TCOM* is a variant of videos with demonstrated efficacy in randomized control trials; therefore, we opted for a more resource- and time-efficient method to test our addition to this set of videos. We used a quasi-experimental design, in which the intervention condition (i.e., *TCOM* and posters) was implemented for 10 months (June 1, 2016-March 31, 2017) and the historical comparison condition (i.e., standard waiting room environment) was the prior 10-month period (August 1, 2015-May 31, 2016). Funding for our study and difficulty in finding eligible HIV clinics precluded use of contemporaneous controls. No patients were recruited and enrolled in the study. Instead, routinely collected data were abstracted electronically from the clinic EMRs for all patients age 18 and older who attended the clinic during the historical comparison period (retrospective data collected June 2016) and during the intervention period (prospective data collected May 2017). Data were de-identified and included dates of index and subsequent outpatient ambulatory medical care (OAMC) visits, sex, gender identity, race, ethnicity, age at index visit, year of HIV diagnosis, date of first ART prescription at the clinic, risks for HIV infection, and HIV viral load laboratory test dates and results.

### Outcome measures

The primary study outcome was ART medication adherence. Adherence was measured by whether viral load suppression (<200 RNA copies/mL) was achieved by 6 months (183 days) after the patient’s index visit; undetectable viral load (<50 RNA copies/mL) also was assessed. Primary outcome data were lab test results in patient EMRs. The secondary study outcomes included treatment initiation and retention in HIV medical care. Treatment initiation was measured by a patient’s receipt of an ART prescription on, before, or within 7 days after, their index visit, as documented by OAMC visits in patient EMRs. Retention in HIV care was measured by an adaptation of the Health Resources and Services Administration’s (HRSA) definition in the Annual Ryan White HIV/AIDS Program Services Report (RSR): “all clients who have had at least 2 or more OAMC visits for any reason at least 90 days apart in the past year divided by all active clients who had had at least one such visit in the past year and are greater than 12 years of age.” [17] HRSA’s definition is restricted to a calendar year; therefore, for HRSA’s calculations, the first OAMC visits in the year must have occurred in the first 8 months (by September 1) of the measurement year in order to give patients an opportunity to meet the retention criteria by year’s end (December 31). However, funding for our study only allowed for a 10-month follow-up. To give patients time to meet retention criteria for our calculations, their first OAMC visit must have occurred in the first 6 months of the study condition period. Our adapted formula comprised patients who came back after 90 days divided by all patients who came in between 1–183 days.

### Statistical analyses

Simplified sample size calculations were performed for rejecting the null hypothesis of equality in the proportions of successes for the study’s primary outcome, assuming equal numbers of patients in each period. These calculations correspond to an analysis using Fisher’s exact test with a two-sided significance level of α = 0.05. We assumed if the true proportions of successes for the two periods were 70% and 77% (historical comparison to intervention, 7 percentage point absolute and 10% relative improvement), then sample sizes of 650 patients already prescribed ART per period would be required to have 80% power to reject the null hypothesis. Allowing for an annual attrition rate of 25%, sample sizes of 1157 patients per period would be required to have 80% power to reject the null hypothesis.

We reviewed EMRs for all patients >17 years of age who had one or more OAMC visits during either the historical comparison or intervention period (100% chart recovery from two sites). Unfortunately, EMR data from the Miami site (all 650 patient records) were incomplete. Miami uses a different EMR system than Huntsville and Atlanta, and important variables were missing or could not be abstracted. Therefore, data from Miami were excluded from our analyses. We analyzed all patients regardless of their exposure to the intervention video.

We computed summary proportions for selected categorical socio-demographic variables (sex/gender, race/ethnicity, age, HIV risk, and time since HIV diagnosis as assessed on index visit date) by site for the comparison and intervention groups (Table 1). We did not statistically test the equivalence of the composition of the comparison and intervention groups because many patients were in both study periods. Simple proportions were used for assessing treatment initiation (Table 2), and a 10-month adaptation of HRSA’s (which administers the Ryan White HIV/AIDS Program) method for retention in care was used for that outcome (Table 3). Time-to-event methods were used for assessing viral load suppression, as a proxy for medication adherence (Tables 4, 5, 6 and 7).

**Table 1:** Characteristics of patients attending HIV clinics in two U.S. cities, August 1, 2015-March 31, 2017.

		Comparison _______				Intervention _______					
Characteristic	Category	Atlanta		Huntsville		Atlanta			Huntsville		
		n	(%)	n	(%)	n		(%)	n		(%)
All patients, N	—	1327		653		1302			721		
Sex/gender	Male	1003 (75.8)		492 (75.3)		998 (76.8)			544 (75.5)		
	Female	299 (22.6)		155 (23.7)		279 (21.5)			166 (23.0)		
	Transgender	21 (1.6)		6 (0.9)		22 (1.7)			11 (1.5)		
Race/ethnicity	Black, non-Hispanic	1204 (90.7)		311 (47.6)		1176 (90.3)			347 (48.1)		
	Hispanic	32 (2.4)		40 (6.1)		29 (2.2)			43 (6.0)		
	White, non-Hispanic	63 (4.8)		291 (44.6)		61 (4.7)			316 (43.8)		
	Other	28 (2.1)		11 (1.7)		36 (2.8)			15 (2.1)		
Age	18–24 years	27 (2.0)		40 (6.1)		53 (4.1)			60 (8.3)		
	25–34 years	468 (35.3)		202 (30.9)		441 (33.9)			225 (31.2)		
	35–87 years	832 (62.7)		411 (62.9)		808 (62.1)			436 (60.5)		
HIV risk	MSM	735 (55.4)		378 (57.9)		741 (56.9)			423 (58.7)		
	Heterosexual	475 (35.8)		234 (35.8)		477 (36.6)			254 (35.2)		
	IDU	31 (2.3)		28 (4.3)		30 (2.3)			24 (3.3)		
	MSM and IDU	20 (1.5)		4 (0.6)		20 (1.5)			5 (0.7)		
	Perinatal	1 (0.1)		4 (0.6)		1 (0.1)			4 (0.6)		
	Transfusion	0 (0.0)		2 (0.3)		3 (0.2)			2 (0.3)		
	Hemophilia	0 (0.0)		1 (0.2)		0 (0.0)			1 (0.1)		
	Not specified	65 (4.9)		2 (0.3)		30 (2.3)			8 (1.1)		
Time since HIV	< 1 year	156 (12.3)		77 (11.8)		135 (10.6)			92 (12.9)		
diagnosis*	1–5 years	483 (38.2)		208 (31.9)		475 (37.2)			217 (30.3)		
	6–10 years	321 (25.4)		161 (24.7)		341 (26.7)			170 (23.7)		
	> 10 years	305 (24.1)		207 (31.8)		327 (25.6)			237 (33.1)		

*Notes*. Other race/ethnicity includes American Indian/Alaska Native, Asian, Pacific Islander/Native Hawaiian, multiple races, and not specified. MSM = men who have sex with men. IDU = injection drug use.

*Time since HIV diagnosis was assessed at index clinic visit date.

**Table 2:** Proportion of antiretroviral therapy (ART) initiation among patients attending HIV clinics in two U.S. cities as measured by ART prescriptions written before or within 7 days after index visit, August 1, 2015-March 31, 2017, by selected patient characteristics.

		Comparison		Intervention		Percentage Point Change (95% CI)		*p*-value
Characteristic	Category	m/n	(%)	m/n	(%)			
All patients, N	—	1980		2023		—		—
Clinic location	Combined	1194/1980	(60.3)	1430/2023	(70.7)	10.4	(7.5, 13.3)	<0.01
	Atlanta	692/1327	(52.1)	788/1302	(60.5)	8.4	(4.6, 12.2)	<0.01
	Huntsville	502/653	(76.9)	642/721	(89.0)	12.2	(8.2, 16.1)	<0.01
Sex/gender	Male	877/1495	(58.7)	1069/1542	(69.3)	10.7	(7.3, 14.1)	<0.01
	Female	301/454	(66.3)	337/445	(75.7)	9.4	(3.5, 15.3)	<0.01
	Transgender	14/27	(51.9)	22/33	(66.7)	14.8	(-10.0, 39.6)	0.30
Race/ethnicity	Black, non-Hispanic	875/1515	(57.8)	1027/1523	(67.4)	9.7	(6.3, 13.1)	<0.01
	Hispanic	43/72	(59.7)	56/72	(77.8)	18.1	(3.2, 32.9)	0.03
	White, non-Hispanic	265/354	(74.9)	318/377	(84.4)	9.5	(3.7, 15.3)	<0.01
	Other	11/39	(28.2)	29/51	(56.9)	28.7	(9.1, 48.3)	0.01
Age	18–34 years	374/737	(50.7)	507/779	(65.1)	14.3	(9.4, 19.3)	<0.01
	35–87 years	820/1243	(66.0)	923/1244	(74.2)	8.2	(4.6, 11.8)	<0.01
HIV risk	Heterosexual	465/709	(65.6)	534/731	(73.1)	7.5	(2.7, 12.2)	<0.01
	MSM	655/1113	(58.9)	818/1164	(70.3)	11.4	(7.5, 15.3)	<0.01
	Other*	74/158	(46.8)	78/128	(60.9)	14.1	(2.6, 25.6)	0.02
Time since HIV	< 1 year	57/233	(24.5)	108/227	(47.6)	23.1	(14.6, 31.6)	<0.01
diagnosis**	1–5 years	440/691	(63.7)	496/692	(71.7)	8.0	(3.1, 12.9)	<0.01
	6–10 years	332/ 482	(68.9)	385/511	(75.3)	6.5	(0.9, 12.0)	0.03
	> 10 years	356/512	(69.5)	434/564	(77.0)	7.4	(2.1, 12.7)	0.01

*Notes*. Other race/ethnicity includes American Indian/Alaska Native, Asian, Pacific Islander/Native Hawaiian, multiple races, and not specified. MSM = men who have sex with men.

*Injection drug use, MSM who inject drugs, hemophilia, transfusion, perinatal, not specified.

**Time since HIV diagnosis was assessed at index clinic visit date.

m = all patients with an ART prescription before their index visit or within 7 days after their index visit (ART initiation). n = total patients in study period.

**Table 3:** Proportion of retention in HIV medical care among patients attending HIV clinics in two U.S. cities as measured by any outpatient ambulatory medical care visit after 3 months of follow-up from their index visit, August 1, 2015-March 31, 2017, by selected patient characteristics.

		Comparison		Intervention		Percentage Point Change (95% CI)		*p*-value
Characteristic	Category	m/n	(%)	m/n	(%)			
All patients, N	—	1980		2023		—		—
Clinic location	Combined	1323/1709	(77.4)	1421/1804	(78.8)	1.4	(-1.4, 4.1)	0.35
	Atlanta	803/1123	(71.5)	849/1153	(73.6)	2.1	(-1.5, 5.8)	0.26
	Huntsville	520/586	(88.7)	572/651	(87.9)	-0.9	(-4.5, 2.7)	0.66
Sex/gender	Male	964/1278	(75.4)	1066/1370	(77.8)	2.4	(-0.9, 5.6)	0.15
	Female	341/405	(84.2)	328/401	(81.8)	-2.4	(-7.6, 2.8)	0.40
	Transgender	15/22	(68.2)	24/30	(80.0)	11.8	(-12.3, 36.0)	0.35
Race/ethnicity	Black, non-Hispanic	984/1302	(75.6)	1044/1353	(77.2)	1.6	(-1.7, 4.8)	0.34
	Hispanic	53/63	(84.1)	61/67	(91.0)	6.9	(-4.4, 18.2)	0.29
	White, non-Hispanic	271/316	(85.8)	285/346	(82.4)	-3.4	(-9.0, 2.2)	0.24
	Other	15/28	(53.6)	31/38	(81.6)	28.0	(5.8, 50.2)	0.03
Age	18–34 years	433/607	(71.2)	519/683	(76.0)	4.8	(-0.1, 9.6)	0.06
	35–87 years	890/1101	(80.8)	902/1121	(80.5)	-0.4	(-3.7, 2.9)	0.83
HIV risk	Heterosexual	506/631	(80.2)	540/660	(81.8)	1.6	(-2.7, 5.9)	0.48
	MSM	732/964	(76.0)	804/1037	(77.5)	1.6	(-2.1, 5.3)	0.43
	Other*	85/114	(74.6)	77/107	(72.0)	-2.6	(-14.3, 9.1)	0.76
Time since HIV	< 1 year	74/125	(59.2)	70/107	(65.4)	6.2	(-6.3, 18.7)	0.35
diagnosis**	1–5 years	496/633	(78.4)	521/660	(78.9)	0.6	(-3.9, 5.1)	0.84
	6–10 years	344/433	(79.4)	375/484	(77.5)	-2.0	(-7.3, 3.4)	0.52
	> 10 years	397/491	(80.9)	452/539	(83.9)	3.0	(-1.7, 7.7)	0.22

*Notes*. Other race/ethnicity includes American Indian/Alaska Native, Asian, Pacific Islander/Native Hawaiian, multiple races, and not specified. MSM = men who have sex with men.

*Injection drug use, MSM who inject drugs, hemophilia, transfusion, perinatal, not specified.

**Time since HIV diagnosis was assessed at index clinic visit date.

m = all patients with an index visit and at least one outpatient ambulatory medical care visit for any reason after 90 days (retained in care). n = all patients who had at least one outpatient ambulatory medical care visit for any reason between 0 and 6 months (183 days) in study period.

**Table 4:** Proportion of medication adherence among patients attending HIV clinics in two U.S. cities as measured by laboratory-confirmed HIV viral suppression (< 200 copies/ml) at 183 days or 6 months, August 1, 2015-March 31, 2017.

		Comparison		Intervention		Percentage Point Change, % (95% CI)		*p*-value
Characteristic	Category	%	(95% CI)	%	(95% CI)			
All patients, N	—	1980		2023		—		—
Clinic location	Combined	56.7	(54.0, 59.4)	62.7	(60.3, 65.1)	6.0	(2.4, 9.6)	<0.01
	Atlanta	41.1	(37.8, 44.7)	58.7	(55.6, 61.8)	17.5	(12.9, 22.2)	<0.01
	Huntsville	77.7	(74.2, 81.1)	69.1	(65.4, 72.8)	-8.6	(-13.7, -3.5)	<0.01

**Table 5:** Proportion of medication adherence among patients attending HIV clinics in two cities as measured by laboratory-confirmed HIV viral suppression (< 200 copies/ml) at 183 days or 6 months, August 1, 2015-March 31, 2017, by subgroups of patient characteristics.

		Atlanta				Huntsville			
		Comparison	Intervention	Percentage Point Change	*p*-value	Comparison	Intervention	Percentage Point Change	*p*-value
Characteristic	Category	% (95% CI)	% (95% CI)	% (95% CI)		% (95% CI)	% (95% CI)	% (95% CI)	
Patients, N	—	1327	1302	—	—	653	721	—	—
Clinic location	—	41.1 (37.8, 44.7)	58.7 (55.6, 61.8)	17.5 (12.9, 22.2)	<0.01	77.7 (74.2, 81.1)	69.1 (65.4, 72.8)	-8.6 (-13.7, -3.5)	<0.01
Sex/gender	Male	40.7 (36.7, 44.9)	57.5 (54.0, 61.1)	16.8 (11.4, 22.2)	<0.01	75.7 (71.5, 79.8)	72.4 (68.1, 76.5)	-3.4 (-9.3, 2.5)	0.26
	Female	42.6 (36.0, 49.8)	61.2 (54.6, 67.8)	18.6 (9.0, 28.1)	<0.01	82.9 (76.3, 88.6)	59.5 (51.5, 67.6)	-23.5 (-33.7, -13.2)	<0.01
	Transgender	33.3 (12.2, 71.8)	70.4 (48.6, 89.2)	37.1 (-0.6, 74.8)	0.05	100 (100, 100)	63.6 (37.3, 88.8)	-36.4 (-64.8, -7.9)	0.01
Race/ ethnicity	Black, non-Hisp	41.8 (38.3, 45.6)	58.3 (55.1, 61.6)	16.5 (11.6, 21.4)	<0.01	74.3 (68.9, 79.4)	65.7 (60.2, 71.2)	-8.6 (-16.3, -0.9)	0.03
	Hispanic	29.4 (13.4, 56.9)	76.0 (56.7, 91.3)	46.6 (18.3, 75.0)	<0.01	87.7 (74.9, 95.8)	69.2 (54.6, 82.7)	-18.5 (-36.5, -0.5)	0.04
	White, non-Hisp	30.4 (18.5, 47.3)	53.6 (39.5, 69.1)	23.3 (2.4, 44.2)	0.03	79.7 (74.5, 84.5)	73.5 (68.0, 78.7)	-6.2 (-13.6, 1.1)	0.10
	Other	61.5 (29.8, 92.4)	66.9 (45.9, 86.3)	5.4 (-37.0, 47.7)	0.80	80.0 (52.5, 96.9)	49.5 (24.3, 81.3)	-30.5 (-70.2, 9.2)	0.13
	Wht+Hisp+Oth	33.6 (23.7, 46.2)	62.2 (51.7, 72.6)	28.6 (13.1, 44.0)	<0.01	80.7 (76.1, 85.0)	72.2 (67.1, 77.0)	-8.6 (-15.3, -1.8)	0.01
Age	18–24 years	25.0 (3.9, 87.2)	50.9 (39.1, 67.6)	25.9 (-19.5, 71.3)	0.26	80.8 (64.3, 92.8)	75.1 (61.5, 86.8)	-5.7 (-25.4, 14.1)	0.58
	25–34 years	46.2 (40.0, 52.8)	52.1 (46.8, 57.6)	5.9 (-2.5, 14.3)	0.17	75.5 (68.8, 81.8)	69.3 (62.6, 75.9)	-6.2 (-15.5, 3.2)	0.20
	35–87 years	39.0 (35.0, 43.3)	62.7 (58.9, 66.5)	23.7 (18.1, 29.4)	<0.01	78.4 (74.1, 82.5)	68.3 (63.5, 73.0)	-10.2 (-16.5, -3.8)	<0.01
HIV risk	MSM	38.2 (33.0, 44.0)	61.9 (56.9, 66.9)	23.7 (16.3, 31.1)	<0.01	81.9 (76.3, 86.9)	65.1 (58.7, 71.4)	-16.8 (-25.1, -8.5)	<0.01
	Heterosexual	43.5 (38.9, 48.5)	56.7 (52.6, 60.9)	13.2 (6.9, 19.6)	<0.01	75.9 (71.1, 80.4)	71.5 (66.6, 76.2)	-4.4 (-11.1, 2.3)	0.20
	Other	39.9 (28.2, 54.2)	56.8 (44.4, 69.8)	16.9 (-1.5, 35.3)	0.07	70.6 (55.6, 84.2)	70.2 (54.3, 84.6)	-0.4 (-22.0, 21.1)	0.97
Time since HIV diagnosis*	< 1 year	39.5 (27.7, 54.1)	51.3 (38.9, 65.0)	11.8 (-7.0, 30.6)	0.22	80.8 (65.2, 92.4)	77.1 (63.0, 88.7)	-3.8 (-23.1, 15.6)	0.70
	1–5 years	40.9 (35.5, 46.7)	59.4 (54.6, 64.3)	18.5 (11.1, 26.0)	<0.01	78.1 (71.8, 83.8)	74.4 (67.9, 80.4)	-3.7 (-12.4, 5.0)	0.40
	6–10 years	38.7 (32.4, 45.8)	51.2 (45.3, 57.4)	12.5 (3.4, 21.5)	0.01	77.9 (71.0, 84.1)	66.1 (58.7, 73.4)	-11.8 (-21.8, -1.9)	0.02
	> 10 years	44.0 (37.6, 51.0)	66.6 (60.8, 72.3)	22.6 (13.8, 31.5)	<0.01	76.5 (70.3, 82.2)	64.3 (57.9, 70.8)	-12.1 (-20.9, -3.3)	0.01

*Notes*. Other race/ethnicity includes American Indian/Alaska Native, Asian, Pacific Islander/Native Hawaiian, multiple races, and not specified. Other HIV risk includes injection drug use, MSM who inject drugs, hemophilia, transfusion, perinatal, and not specified. Hisp = Hispanic. MSM = men who have sex with men. N = total patients in study period. Oth = Other. Wht = White.

*Time since HIV diagnosis was assessed at index clinic visit date.

**Table 6:** Proportion of medication adherence among patients attending HIV clinics in two U.S. cities as measured by laboratory-confirmed undetectable HIV viral load (< 50 copies/ml) at 183 days or 6 months, August 1, 2015-March 31, 2017.

		Comparison		Intervention		Percentage Point Change, % (95% CI)		*p*-value
Characteristic	Category	%	(95% CI)	%	(95% CI)			
All patients, N	—	1980		2023		—		—
Clinic location	Combined	36.7	(34.2, 39.4)	40.6	(38.2, 43.0)	3.8	(0.3, 7.4)	0.03
	Atlanta	9.7	(7.8, 12.0)	24.9	(22.3, 27.7)	15.2	(11.8, 18.6)	<0.01
	Huntsville	73.4	(69.6, 77.0)	65.8	(61.9, 69.6)	-7.6	(-12.9, -2.3)	0.01

**Table 7:** Proportion of medication adherence among patients attending HIV clinics in two cities as measured by laboratory-confirmed undetectable HIV viral load (< 50 copies/ml) at 183 days or 6 months, August 1, 2015-March 31, 2017, by subgroups of patient characteristics.

		Atlanta				Huntsville			
		Comparison	Intervention	Percentage Point Change CI)	*p*-value	Comparison	Intervention	Percentage Point Change	*p-value*
Characteristic	Category	% (95% CI)	% (95% CI)	% (95%		% (95% CI)	% (95% CI)	% (95% CI)	
Patients, N	—	1327	1302	—	—	653	721	—	—
Clinic location	—	9.7 (7.8, 12.0)	24.9 (22.3, 27.7)	15.2 (11.8, 18.6)	<0.01	74.0 (69.6, 77.0)	65.8 (61.9, 69.6)	-7.6 (-12.9, -2.3)	0.01
Sex/gender	Male	7.7 (5.7, 10.2)	23.9 (21.0, 27.1)	16.3 (12.5, 20.0)	<0.01	71.3 (66.8, 75.6)	68.5 (64.1, 72.8)	-2.8 (-9.0, 3.4)	0.37
	Female	16.1 (11.7, 22.0)	28.0 (22.5, 34.6)	11.9 (4.0, 19.8)	<0.01	78.8 (71.8, 85.1)	57.4 (49.3, 65.7)	-21.5 (-32.1,-10.8)	<0.01
	Transgender	0.0 (0.0, 33.6)	28.9 (13.2, 56.3)	28.9 (0.5, 57.3)	0.05	100 (100, 100)	63.6 (37.3, 88.8)	-36.4 (-64.8, -7.9)	0.01
Race/ ethnicity	Black, non-Hisp	9.9 (7.9, 12.4)	25.0 (22.4, 28.0)	15.1 (11.6, 18.7)	<0.01	69.9 (64.3, 75.4)	61.6 (55.9, 67.3)	-8.3 (-16.3, -0.3)	0.04
	Hispanic	0.0 (0.0, 24.7)	19.0 (7.6, 43.1)	19.0 (-2.5, 40.6)	0.08	90.8 (78.6, 97.5)	66.7 (52.0, 80.7)	-24.1 (-41.8, -6.5)	0.01
	White, non-Hisp	10.2 (4.0, 25.0)	25.7 (15.1, 41.7)	15.5 (-0.7, 31.8)	0.06	76.0 (70.5, 81.1)	71.0 (65.4, 76.4)	-5.0 (-12.6, 2.7)	0.20
	Other	10.0 (1.5, 52.7)	24.3 (10.8, 49.2)	14.3 (-12.1, 40.7)	0.29	76.7 (47.1, 96.4)	59.6 (32.6, 87.5)	-17.1 (-58.5, 24.3)	0.42
	Wht+Hisp+Oth	7.5 (3.2, 17.2)	23.7 (15.9, 34.3)	16.1 (5.0, 27.3)	<0.01	77.5 (72.6, 82.1)	69.4 (64.2, 74.5)	-8.1 (-15.1, -1.1)	0.02
Age	18–24 years	0.0 (0.0, 70.8)	13.0 (5.6, 28.7)	13.0 (-30.8, 56.8)	0.56	73.7 (56.7, 88.1)	68.2 (53.9, 81.6)	-5.5 (-27.2, 16.3)	0.62
	25–34 years	5.5 (3.2, 9.2)	18.5 (14.8, 23.2)	13.1 (8.0, 18.2)	<0.01	70.8 (63.8, 77.6)	65.1 (58.1, 71.9)	-5.8 (-15.6, 4.0)	0.25
	35–87 years	11.7 (9.2, 14.7)	29.1 (25.7, 32.8)	17.4 (12.9, 21.9)	<0.01	74.4 (69.8, 78.8)	65.8 (60.9, 70.6)	-8.7 (-15.3, -2.0)	0.01
HIV risk	MSM	14.4 (10.9, 18.8)	29.6 (25.2, 34.5)	15.2 (9.1, 21.3)	<0.01	77.1 (71.0, 82.6)	62.0 (55.5, 68.5)	-15.1 (-23.8, -6.3)	<0.01
	Heterosexual	6.6 (4.5, 9.4)	20.9 (17.8, 24.5)	14.3 (10.2, 18.5)	<0.01	72.0 (67.0, 76.8)	67.6 (62.7, 72.5)	-4.3 (-11.4, 2.7)	0.22
	Other	7.3 (2.8, 18.5)	33.9 (23.3, 47.6)	26.6 (12.6, 40.6)	<0.01	65.4 (50.3, 80.0)	70.2 (54.3, 84.6)	4.8 (-17.1, 26.7)	0.67
Time since HIV diagnosis*	< 1 year	5.8 (1.9, 16.8)	19.8 (11.6, 32.5)	14.0 (2.0, 26.0)	0.02	72.2 (55.5, 86.8)	69.9 (55.2, 83.4)	-2.3 (-24.2, 19.5)	0.83
	1–5 years	5.4 (3.4, 8.7)	23.2 (19.4, 27.7)	17.8 (12.9, 22.7)	<0.01	74.1 (67.5, 80.2)	69.3 (62.6, 75.8)	-4.8 (-14.0, 4.4)	0.31
	6–10 years	11.8 (8.1, 17.1)	20.2 (15.8, 25.6)	8.4 (1.8, 15.0)	0.01	75.5 (68.3, 82.0)	63.8 (56.3, 71.3)	-11.6 (-21.9, -1.4)	0.03
	> 10 years	14.7 (10.6, 20.2)	33.2 (27.9, 39.3)	18.5 (11.1, 26.0)	<0.01	71.0 (64.5, 77.2)	62.3 (56.1, 69.1)	-8.4 (-17.6, 0.7)	0.07

*Notes*. Other race/ethnicity includes American Indian/Alaska Native, Asian, Pacific Islander/Native Hawaiian, multiple races, and not specified. Other HIV risk includes injection drug use, MSM who inject drugs, hemophilia, transfusion, perinatal, and not specified. Hisp = Hispanic. MSM = men who have sex with men. N = total patients in study period. Oth = Other. Wht = White.

*Time since HIV diagnosis was assessed at index clinic visit date.

Planned primary analyses included outcomes of ART prescription within 7 days of index visit (Table 2), follow-up OAMC visits during a study period (Table 3), and having suppressed viral load (Tables 4 and 5). Subjects had one index visit per condition, and visits during one condition could not be used for measurements in the other condition. For example, an OAMC visit early in the intervention period was not counted toward retention for the comparison period. A planned secondary analysis was having an undetectable viral load (Tables 6 and 7) as an outcome. For binary outcomes, we computed proportions in the comparison and intervention arms, the percentage point change with approximate 95% confidence intervals, and *p*-values for Fisher’s exact test. For time-to-event outcomes, we computed Kaplan-Meier proportions at 183 days (6 months) in the comparison and intervention arms, the percentage point change with approximate 95% confidence intervals, and *p*-values based on the normal approximation.

We performed planned subgroup analyses to assess whether intervention effects varied by clinic and by selected categorical socio-demographic variables (sex/gender, race/ethnicity, age, HIV risk, and time since HIV diagnosis as assessed on index visit date). All analyses were performed using SAS statistical software (version 9.4, SAS Institute, Inc., Cary, NC) and were conducted by one author (CBB). All statistical tests were two-tailed. We did not adjust for the overlap between the comparison and intervention groups, which tends to give conservative significance tests assuming positive correlations between outcomes.

## Results

**Fig 1:**
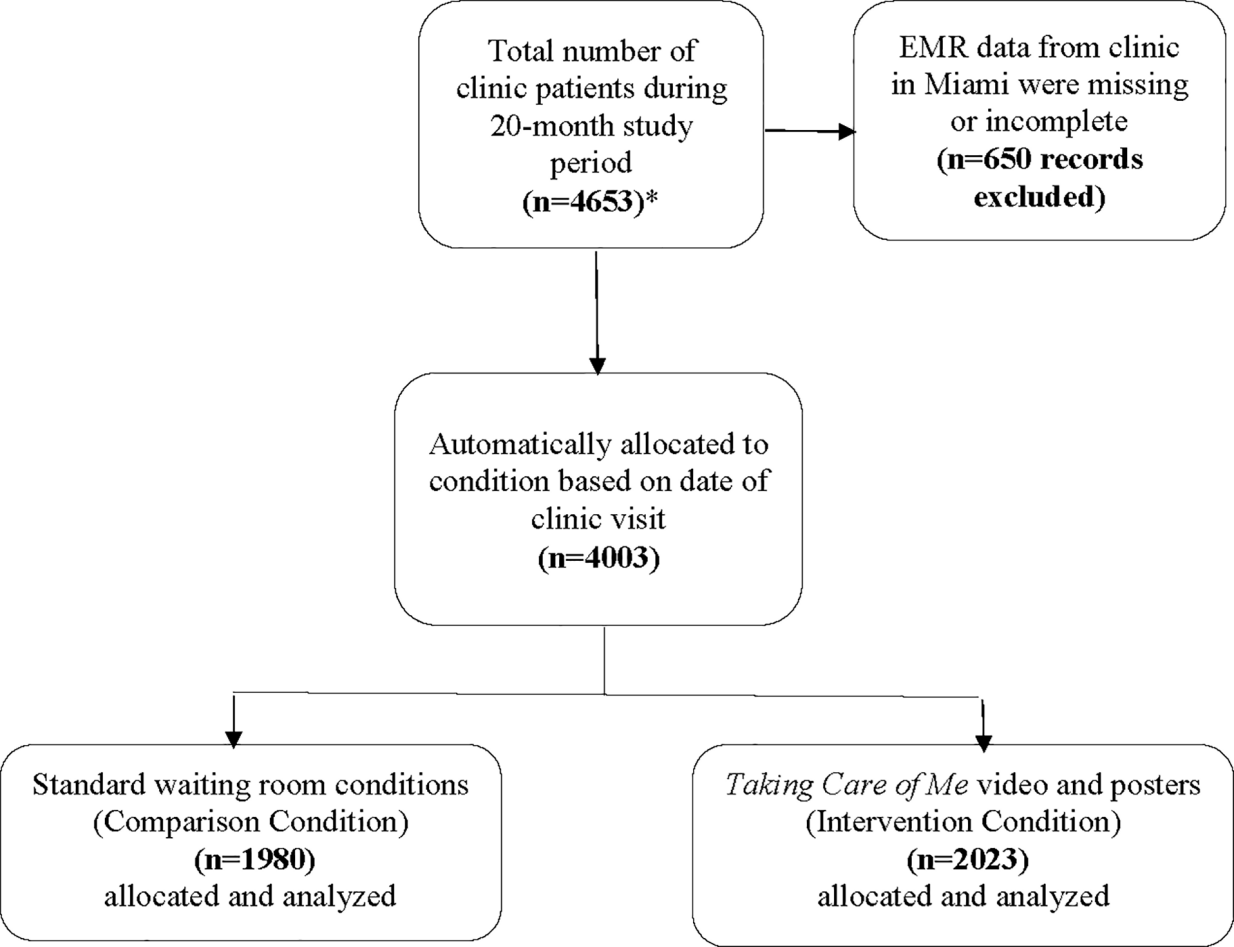
Flow diagram of Taking Care of Me study subjects, August 1, 2015 –March 31, 2017. *The 10-month study period for August 1, 2015 to May 31, 2016 was the historical comparison condition. The 10-month study period from June 1, 2016 to March 31, 2017 was the intervention condition where all patients attending two HIV clinics were exposed to the *Taking Care of Me* video.

There were a total of 4003 patients across both study conditions (Atlanta = 2629, Huntsville = 1374) (Fig 1, S2 Appendix, Table 1). Of the patients in Atlanta 1706 were in one study condition, and 923 (54.1%) were in both conditions. Of the patients in Huntsville 794 were in one study condition, and 580 (73.0%) were in both conditions. Patient sex, gender identity, and HIV risks were similar across sites and conditions. Compared to Atlanta, Huntsville had slightly more (4 percentage points) younger patients (18–24 year category) and somewhat more (8 percentage points) patients who received their HIV diagnosis over 10 years ago. The main difference between sites was race and ethnicity. Atlanta’s clinic population was 90% black, while Huntsville’s was 48% black and 44% white. No adverse events were reported during the study.

For HIV treatment initiation we found a significant (p < 0.01) overall increase of 10.4 percentage points between study periods and significant intervention effects at both sites. (Table 2) Significant but differential intervention effects were observed for sex, race/ethnicity, age, HIV risk exposure, and time since HIV diagnosis. Treatment initiation increased among males (10.7 percentage points) and females (9.4 percentage points) between study periods (both p < 0.01). Significant increases were observed among blacks (9.7 percentage points, p < 0.01), Hispanics (18.1 percentage points, p = 0.03), whites (9.5 percentage points, p < 0.01), and persons of ‘other’ race or ethnicity (28.7 percentage points, p = 0.01). There were significant increases (p < 0.01) among persons 18–34 years of age (14.3 percentage points) and persons 35–87 years of age (8.2 percentage points). Treatment initiation increased between study periods among persons reporting heterosexual behavior (7.5 percentage points, p < 0.01), male-to-male sex (11.4 percentage points, p < 0.01), and persons reporting ‘other’ risk behaviors (14.1 percentage points, p = 0.02). Significant increases were found among those diagnosed <1 year (23.1 percentage points, p < 0.01), 1–5 years (8.0 percentage points, p < 0.01), 6–10 years (6.5 percentage points, p = 0.03), and >10 years (7.4 percentage points, p = 0.01) ago who were exposed to the intervention.

Differences in retention in HIV care, although observed in the anticipated direction for both sites combined (1.4 percentage point increase), were not statistically significant and did not differ substantially by site (Table 3). Retention increased 28.0 percentage points between study periods for patients in the ‘other’ racial category. Although the number of patients in this sub-group was small, this difference was statistically significant (p = 0.03). No statistically significant differences were observed when examined across sex/gender, remaining race/ethnicity categories, age, HIV risk, or time since HIV diagnosis.

Viral suppression was the primary measure of medication adherence. At Huntsville and Atlanta combined, viral suppression improved 6.0 percentage points (p < 0.01) between study periods. (Table 4) However, viral suppression in Atlanta increased by 17.5 percentage points (p < 0.01), but decreased in Huntsville by 8.6 percentage points (p < 0.01). In Atlanta, all of the percentage point changes for subgroups were increasing between study periods. Significant increases were observed for patients who were male or female; black, Hispanic, or white; between 35–87 years old; had heterosexual or male-to-male sexual risk; and were diagnosed one or more years ago. (Table 5) Conversely, in Huntsville, all of the percentage point changes for subgroups were decreasing between study periods. Significant decreases were observed for patients who were female or transgender; black or Hispanic; between 35–87 years old; had male-to-male sexual risk; and were diagnosed six or more years ago.

Similar findings were observed with respect to the secondary measure of laboratory-confirmed undetectable viral load. At both sites combined, undetectable viral load improved 3.8 percentage points (p = 0.03) between study periods. (Table 6) The sites again had divergent results. Undetectable viral load in Atlanta improved by 15.2 percentage points (p < 0.01), but dropped in Huntsville by 7.6 percentage points (p = 0.01). Again in Atlanta, all of the percentage point changes for subgroups were increasing between study periods. (Table 7) Significant increases were observed for patients who were male or female; black; between 25–34 or 35–87 years old; for any transmission risk category; and for any time since diagnosis. Conversely, in Huntsville, all of the percentage point changes for subgroups were decreasing between study periods. Significant decreases were observed for patients who were female or transgender; black or Hispanic; between 35–87 years old; had male-to-male sexual risk; and were diagnosed 6–10 years ago.

## Discussion

In our evaluation of the *TCOM* video, we found a significant overall increase in the percentage of treatment initiators among patients who visited the sites while the video played and posters were displayed (intervention condition), compared to patients who visited without the video and posters (comparison condition). The overall positive change in treatment initiation also was observed in sub-group analyses of demographic (i.e., sex, race/ethnicity, age) and behavioral characteristics (i.e., HIV risk, time since HIV diagnosis). The nature of the study design allows us to draw conclusions about association but not causation. Given that *TCOM* featured multiple vignettes that modeled PWH of diverse racial/ethnic backgrounds and sexual orientations, it is not surprising that the video showed evidence of changing behavior among a heterogeneous patient population. However, the overall effects for retention in HIV care were statistically non-significant. Medication adherence, measured by viral suppression and undetectable viral load, was significantly greater during the intervention period than the comparison period in Atlanta and both sites combined. Other co-occurring factors may have contributed to observed results, although their potential effect is unclear. For example, the findings from the Strategic Timing of Antiretroviral Therapy (START) study, which showed the benefit of early ART initiation, were published in August 2015. [1] START’s results were widely known prior to publication and likely integrated in clinical practice before our study’s comparison period. Also, HIV treatment guideline recommendation to initiate ART for all PWH was upgraded to the highest level (i.e., A1) in January 2016. [18] Finally, health insurance coverage increased nationwide during the study period; however, most PWH are Medicaid-eligible. Alabama and Georgia are not Medicaid expansion states, so increased national Medicaid coverage likely had no impact on the numbers of PWH attending the study clinics.

Unlike Atlanta, Huntsville’s adherence results were negative. The reason is unclear. We were unable to explore influences of socioeconomic status, educational attainment, or health insurance coverage because the study did not collect these EMR data. Stratifying by race/ethnicity, age, HIV risk, and time since HIV diagnosis did not explain the difference between sites. Sub-group analyses of adherence in Huntsville showed negative results across all groups, suggesting systemic (e.g., clinic hours) or environmental (e.g., local policies) factors at work. We contacted the Huntsville clinic to inquire about any such changes between study conditions. The Huntsville clinic confirmed there had been a 23.5% increase in the number of new patients during the intervention period but reported no environmental, political, policy, or other changes in the clinic or Huntsville that could have contributed to the observed intervention effects (S3 Appendix). It is highly probable that the Huntsville clinic patients who were new to HIV treatment in the intervention condition did not have time to become virally suppressed during the study measurement period. If we had used a less stringent medication adherence measure (e.g., any viral load suppression within study period), the results may have been higher. Ceiling effects also may have contributed to the between-site difference. Huntsville had 77.7% suppression and 73.4% undetectable proportions during the comparison period; these proportions were only 41.1% and 9.7%, respectively, in Atlanta. Thus, Huntsville had less room for improvement.

The results presented in this paper extend the findings of Warner et al. (2008) [13] by showing that a video-based intervention focused on HIV treatment can be effective at improving HIV clinical outcomes. Having an effective tool that can be easily delivered in a waiting room affords a high degree of audience-targeting with relatively low effort. As an effective approach to supporting improved HIV clinical outcomes, this intervention has the potential to be widely disseminated to HIV clinics and other appropriate settings. Additionally, it is reasonable to expect that the acceptability among patients and clinics seen with *SITC* [19] would be true of *TCOM*.

Video as an intervention tool continues to be prominent in current public health approaches. [20] *TCOM* represents the first stand-alone video-based intervention we are aware of that is effective at improving HIV-related health outcomes for PWH. The relative overall change in treatment initiation (i.e., starting a new behavior) for *TCOM* is comparable to the level of change in incident STDs (i.e., resulting from adopting new behaviors) for *SITC* (10%). [13] Although video-based intervention effect sizes may be modest when compared to those achieved from more resource-intensive interventions, video-based interventions deliver their content consistently and can be scaled more efficiently, because they are rapid to disseminate, easy to implement, and inexpensive to sustain. For example, 260 agencies initiated *V/V* in the first 20 months of its availability [21], and over 2100 clinics requested *SITC* in its first 15 months. [19] *TCOM* has the potential to be an important addition to the *V/V* and *SITC* set of videos. The time and resources required for rigorous evaluation are barriers to developing and evaluating new videos, and changes in societal norms and advances in HIV prevention and treatment may influence a video’s continued relevance. Given the relative scarcity of public health resources, the trade-offs between faster dissemination and lengthy evaluation should be considered. By forgoing intensive evaluation of videos that follow successful models, new prevention videos could be made available sooner. This could likely have a significant positive impact on the health of PWH, reduce future health care costs, and help achieve national HIV prevention, care, and treatment goals.

Our study suggests that there were significant intervention effects among specific sub-groups for treatment initiation and medication adherence outcomes. For treatment initiation, most demographic and behavioral characteristic groups appeared to have benefited from *TCOM* exposure. It is plausible that the observed intervention effects are due partly to the video’s embedded prevention messages and its appeal to a wide patient audience. However, the intervention may have had an indirect effect if clinicians watched the video and learned how to effectively dialogue with patients about the benefits of early treatment. For both measurement levels of medication adherence, males, blacks, and older patients appeared to have benefited from exposure to *TCOM*. The significant intervention effects for both adherence measures seen among Atlanta, but not Huntsville, patients are difficult to explain. In addition to a possible ceiling effect, it is probable that Huntsville’s simultaneous increases in patient population and treatment initiation temporarily increased clinic-wide viral load levels. Retention in care is a difficult outcome to measure in a standard way. HRSA’s definition needed to be adapted for this study design, and the study’s 10-month intervention period may not have been enough time to see the full effects of the intervention.

### Study limitations

The study had a number of limitations. First, it was not possible to randomly assign patients to one or the other study condition; therefore, the findings may be subjected to bias. Second, the quasi-experimental study design is subject to secular trends (e.g., health insurance enrollment periods). Third, we did not measure exposure to the video, so we do not know whether or not patients paid attention to the video or saw it multiple times. Fourth, Huntsville played the Spanish-captioned video uninterrupted, while Atlanta played the English-captioned video with minor interruptions and some competition from another television. Finally, the sample size was affected by the exclusion of Miami’s data.

Our study design also had several strengths, especially with regard to rapid intervention development and evaluation to promote improved HIV outcomes. Similar to our previous video-based intervention studies in STD clinics [10, 11, 13], this evaluation was conducted under clinic conditions. By integrating *TCOM* into the waiting room environment of two HIV clinics, we were able to evaluate intervention effectiveness in increasing treatment initiation and medication adherence among a relatively large clinic population of PWH. Our evaluation used medical outcomes and routinely collected EMR data rather than behavioral outcomes and self-reported data. Consequently, our overall treatment initiation and medication adherence findings are likely generalizable to other HIV-infected patients at these and other clinics with similar patient characteristics. Similar to our previous work [13], our study design included all clinic patients, thereby avoiding threats to external validity (e.g., low or differential participation rates) inherent in studies using only willing participants. Given that patients were not actively enrolled, they did not perceive the video as part of a study, and biases associated with active study participation were avoided.

We found that a brief video-based behavioral intervention in a clinic setting was effective at improving HIV-related outcomes among PWH. Due to its ease of delivery in a waiting room setting and low cost to sustain, it is important for HIV clinics and other healthcare settings serving PWH to consider integrating the *TCOM* video into standard clinical practice.

## Supporting information

S1 Appendix Study protocol. (DOCX)

S2 Appendix TREND checklist. (PDF)

S3 Appendix Huntsville clinic communication. (PDF)

S1 Dataset Taking Care of Me de-identified study data file. (ZIP)

## Abbreviations

ART: Antiretroviral therapy; the daily use of a combination of HIV medicines to treat HIV infection.
EMR: Electronic medical record.
HIV: Human immunodeficiency virus; the virus that causes Acquired Immunodeficiency Syndrome.
HRSA: Health Resources and Services Administration; an agency of the U.S. Department of Health and Human Services, which is the primary federal agency for improving health care to people who are geographically isolated, economically or medically vulnerable
IRB: Institutional review board; a formally appointed committee that is responsible for the ethical review and oversight of research involving human participants.
OAMC: Outpatient ambulatory medical care; the provision of professional diagnostic and therapeutic services rendered by a physician, physician's assistant, clinical nurse specialist, or nurse practitioner in an outpatient setting
PWH: Persons with HIV.
SITC: *Safe in the City;* an intervention video designed for use in STD clinic waiting rooms.
START: Strategic Timing of Antiretroviral Therapy; a study that showed the benefit of early ART initiation.
STD: Sexually transmitted disease.
TCOM: *Taking Care of Me*, an intervention video designed for use in HIV clinic waiting rooms
V/V: *Video Opportunities for Innovative Condom Education and Safer Sex/Video y Oportunidades Innovadoras acerca de los Condones y la Educación del Sexo más Seguro* (also known by its acronym *VOICES/VOCES*); a video-based, group-level intervention designed for use by STD clinics
