# Supplementary material for: Effects of a brief video intervention on treatment initiation and adherence among patients attending human immunodeficiency virus treatment clinics
Source: PLoS One. 2018 Oct 5;13(10):e0204599. doi: 10.1371/journal.pone.0204599 (PMC6173379; doi:10.1371/journal.pone.0204599)
Supplement: S1 Appendix — (DOCX) [file pone.0204599.s001.docx]

**IRB PROTOCOL**

**A Waiting Room-Delivered Video to Enhance Antiretroviral Therapy Readiness, Adherence, and Retention in Care for Minority Persons Living With HIV Infection**

**Sentient Research**

**January 20, 2016**

# TABLE OF CONTENTS

| I. PROJECT OVERVIEW | 3 |
| --- | --- |
|  |  |
| II. INTRODUCTION | 4 |
|  |  |
| III. PROCEDURES/METHODS | 6 |
|  |  |
| *A. DESIGN* | 6 |
|  |  |
| *B. STUDY POPULATION* | 6 |
|  |  |
| *C. VARIABLES/INTERVENTIONS* | 8 |
|  |  |
| *D. DATA HANDLING AND ANALYSIS* | 10 |
|  |  |
| *E. HANDLING OF UNEXPECTED FINDINGS AND ADVERSE EVENTS* | 12 |
|  |  |
| *F. SHARING STUDY RESULTS* | 13 |
|  |  |
| IV. HUMAN SUBJECTS PROTECTIONS | 14 |
|  |  |
| *A. INFORMED CONSENT* | 14 |
|  |  |
| *B. RISKS & BENEFITS* | 14 |
|  |  |
| *C. PRIVACY & CONFIDENTIALITY* | 14 |
|  |  |
| V. REFERENCES | 15 |
|  |  |
| VI. APPENDIX MATERIALS | 16 |

**I. PROJECT OVERVIEW**

**Title: A Waiting Room-Delivered Video to Enhance Antiretroviral Therapy Readiness, Adherence, and Retention in Care for Minority Persons Living With HIV Infection**

**Protocol summary:**

The purpose of this project is to beta-test a brief waiting room video intervention that promotes early initiation of antiretroviral treatment among treatment-naïve HIV-positive patients, adherence to antiretroviral treatment and retention in care among HIV-positive patients currently on therapy, sexual risk reduction tailored to HIV-positive persons, and patient-initiated discussion of these topics with their health providers. The video is currently being created, and will be ready for beta-testing by June 1, 2016. Results of the beta-testing will be used to refine and improve the video before dissemination to HIV/AIDS treatment facilities nationally. This video project is being funded by the Centers for Disease Control and Prevention, Division of HIV/AIDS Prevention (DHAP).

The overall goal of this study is to beta-test the waiting room video in three HIV/AIDS treatment facilities in different US jurisdictions with high AIDS prevalence. These results will be used to refine the video and to increase its effectiveness among minority persons living with HIV infection. The beta-testing will consist of: 1) unobtrusive observations of waiting room patients for one day at each facility and 2) de-identified, existing patient medical data abstracted from each facility’s electronic medical record (EMR) system to investigate outcomes relevant to HIV medication adherence and retention in care.

One main hypothesis will be tested in a systematic observation design:

Hypothesis 1: Exposure to the video will elicit favorable reactions from patients as evidenced by their paying attention to the video and other positive responses, such as smiling.

Three main hypotheses will be tested in a historical comparison/intervention design:

Hypothesis 2: Exposure to the video will contribute to a greater proportion of HIV-positive persons currently on treatment who adhere to their HIV medication regime.

Hypothesis 3: Exposure to the video will contribute to earlier initiation of antiretroviral therapy (ART) among treatment naïve HIV-positive persons.

Hypothesis 4: Exposure to the video will increase retention in care among HIV-positive persons in care.

**Roles and Responsibilities:**

Aaron Plant, MPH, Sentient Research – (PI and Beta-Test Manager) As PI, Mr. Plant will provide day-to-day oversight of the study, including implementation, data collection coordination, and oversight of the data manager. As beta-test manager, Mr. Plant will be the observer responsible for conducting the systematic observations in participating facilities and will compile and clean all observational data. He will also consult regularly with the participating facilities’ managers and EMR data staff and with the CDC staff.

Marjan Javanbakht, PhD, Sentient Research – (Data Manager) will be responsible for data management, data security, compiling data, and transferring data via an encrypted USB drive to the CDC for analysis.

EMR data staff at each clinic, TBD – will be responsible for de-identifying patient medical data, transferring data onto an encrypted USB drive, and mailing the USB drive to the data manager.

Mary Neumann, PhD, Centers for Disease Control and Prevention – (Contract Officer’s Representative) will be responsible for the monitoring and oversight of the technical and programmatic aspects of the contract and for receiving and analyzing the data. She will consult regularly with the project PI. Dr. Neumann will oversee the dataset received from the data manager and the publications and manuscripts prepared from project data.

Andrew Margolis, MPH, Centers for Disease Control and Prevention – (Advisor) will be responsible for advising on the technical and programmatic aspects of the video development and data collection and analyses. Mr. Margolis will participate in preparation of publications and manuscripts from project data.

Craig Borkowf, PhD, Centers for Disease Control and Prevention – (Advisor) will be responsible for advising on the statistical aspects of collecting and interpreting the data. Dr. Borkowf will participate in data analyses and in preparation of publications and manuscripts from project data.

***Investigators/collaborators/funding mechanism(s)/Federalwide Assurance numbers/ “engagement in research” status:***

| Name | Funding Mechanism | Funding Numbers | Federalwide Assurance Numbers* | “Engagement” in Research** |
| --- | --- | --- | --- | --- |
| Aaron Plant, MPH / Sentient Research | Contract | #200-2013-57577 |  | No |
| Marjan Javanbakht, PhD / Sentient Research | Contract | #200-2013-57577 |  | No |

**II. INTRODUCTION**

**Literature review/current state of knowledge about project topic:**

HIV antiretroviral therapy, when initiated early and taken as directed, has been shown to have substantial health benefits for people with HIV. HIV positive persons who begin taking medication early have better outcomes in terms of opportunistic infections; patients with well managed HIV and resulting low viral loads are much less likely to transmit HIV to their sex partners.^1^ HIV treatment guidelines issued by the National Institutes of Health suggest that HIV treatment should begin as soon as the patient is diagnosed with HIV,^2^ but getting patients into treatment and maintaining an adherence to HIV antiretroviral therapy continues to be a challenge.

Indeed, despite the documented benefits, only 37% of people with HIV are retained in care, only 33% are prescribed antiretroviral therapy, and only 25% have their HIV under control.^3^ Of particular concern, just 21% of African-Americans have their HIV under control.^3^ Effective and culturally competent interventions are needed to increase the number of people with managed HIV. The CDC’s *Compendium of HIV Prevention Interventions with Evidence of Effectiveness* has identified 10 interventions for improving medication adherence among HIV-positive persons; 35 sexual or drug risk reduction interventions for heterosexual adults and 18 for high-risk youth; and no interventions for improving retention in HIV care.^4^ All but one of these interventions requires a trained facilitator to deliver it. Facilitator-led interventions delivered in scheduled individual- or small group-level formats at specific locations require significant provider resources to implement and substantial client time commitments. Behavioral interventions that are time- or resource-intensive may be difficult to sustain or scale up to a level required to have an impact and benefit everyone who needs them. Therefore, low cost, sustainable interventions such as waiting room videos are very promising ways to effect positive behavior change with regard to antiretroviral therapy uptake, adherence, and prevention for large numbers of HIV-positive persons very cost-effectively and on a large scale.

**Justification for study:**

Enhancing the impact of prevention and care services for people living with HIV/AIDS and reducing HIV-related health disparities and inequities among minority persons living with HIV/AIDS are public health priorities. Key HIV prevention strategies include promoting HIV treatment readiness, anti-retroviral therapy (ART) adherence, retention in care, and safer sexual behaviors among people living with HIV/AIDS. There is a significant need for interventions that incorporate these strategies and that can be brought to scale quickly, provide broad coverage, and be sustained at low cost. A stand-alone video-based intervention can engage large numbers of patients without relying on trained facilitators. Video-delivered interventions are 1) low cost to produce, 2) low staff effort to implement, 3) high impact, 4) brief, 5) easily sustainable, and 6) quickly scalable.

The project will develop, beta-test, and refine a brief “soap opera” style video with three interwoven vignettes that incorporate key prevention messages aimed at increasing treatment readiness, ART adherence, retention in care, and partner protection among persons living with HIV/AIDS who attend HIV/AIDS treatment facilities. The video will be designed for use on television monitors in waiting rooms or exam rooms and on other modalities (e.g., Smartphones and tablets). The video will have English and Spanish captions to increase its usability. The waiting room video has the potential for widespread impact for people with HIV and their sex partners.

**Intended/potential use of study findings:**

The purpose of the study is to beta-test and subsequently refine and improve the video to increase its effectiveness among HIV-positive persons in medical care settings. Publications and presentations will be prepared for academic and non-academic audiences, with the intent of informing future research and prevention efforts in patient retention and medication adherence. The final, refined video will be made available for national dissemination and use.

**Study design:**

***Location(s):*** The three HIV/AIDS treatment facilities where the beta-testing will take place are: 1) HIV Primary Care Clinic, Atlanta, GA; 2) AIDS Action Coalition, Huntsville, AL; and 3) the Borinquen Medical Center, Miami, FL.

The waiting room at the Borinquen Medical Center is not exclusively for HIV-positive patients; therefore, the video will be shown across the street in the Medical Center’s HIV case management office. The systematic observations will be conducted in the waiting room of the case management office, and patient medical data will be abstracted from Borinquen Medical Center’s EMR system.

***Objectives: The objectives of this investigation are:***

1. Identifying points in the video that need refinement by unobtrusively observing whether certain video segments or scenes elicit a positive or negative response or have better or worse patient engagement among HIV-positive waiting room patients of various ages, genders, and race/ethnicities.
2. Assessing the impact of the video intervention by comparing HIV-positive patient outcomes relevant to HIV medication initiation and adherence, retention in HIV medical care, and partner protection for the 10-month period before the video intervention with the same outcomes for the 10-month period during the video intervention.

***Hypotheses:***

1. Exposure to the video will elicit favorable reactions from patients as evidenced by their paying attention to the video and other positive responses, such as smiling.
2. Exposure to the video will contribute to a greater proportion of HIV-positive persons currently on treatment who adhere to their HIV medication regimen.
3. Exposure to the video will contribute to earlier initiation of antiretroviral therapy (ART) among treatment naïve HIV-positive persons.
4. Exposure to the video will increase retention in care among HIV-positive persons in care.
5. Exposure to the video will decrease sexually transmitted disease (STD) incidence among HIV-positive persons in care.

***General approach:*** The approach will be developmental. Both the observation and the review of de-identified patient medical data are meant to identify areas of the video that need to be refined or improved to optimize its effectiveness.

**III. PROCEDURES/METHODS**

***A. DESIGN***

***How study design addresses hypotheses and meets objectives:*** The observational data will address Objective 1 and allow us to answer Hypothesis 1. By systematically and unobtrusively observing patients for one full day in each facility’s waiting room, we will identify parts of the video that are less engaging to the patients in the waiting room. Less engaging segments will be refined. The review of medical data will address Objective 2 and allow us to answer Hypotheses 2, 3, 4, and 5. This de-identified medical data will show whether HIV/AIDS treatment facility patients experienced overall increases in ART initiation, HIV medication adherence, and clinic visits and, possibly, overall decrease in STD incidence during the 10-month intervention period compared to the 10 months prior.

***Audience and stakeholder participation:***  The primary audience for the video will be HIV-positive patients in each of the beta-test facilities. Stakeholders will include the project team responsible for creating the video and overseeing the beta-testing, staff at each beta-test facility, and CDC staff. Members of the project team responsible for creating the video include technical consultants who are clinic managers and technical consultants who are persons living with HIV. The beta-test facility staff will participate in the study by showing the video, contributing de-identified medical data, and allowing a beta-tester to do one day of observation in the facility’s waiting room. They also can share their experience of showing the video in their waiting room with the project team. CDC staff will analyze the data and make recommendations for refinement of video segments. HIV-positive patients are not enrolled in the study, and will not be approached at any time by the observer. Therefore, they contribute to the study through their reactions to the video and through their medical data collected from the facilities’ EMRs.

***Study timeline:***

The intervention period will begin in June 2016 and go through March 2017. Prior to beginning the intervention, data collection will be pilot tested in each facility. In June of 2016, each facility will provide 10 months of retrospective de-identified medical data. An interim collection of the data will occur in December 2016, and the final data will be collected from the facilities in April 2017. The systematic observations will occur within the first two months of the intervention period.

***B. STUDY POPULATION***

***Description and source of study population and catchment area:***

The study population will be adult HIV-positive patients at three HIV/AIDS treatment facilities in different US jurisdictions. All adults who are HIV-positive patients in the participating HIV/AIDS treatment facilities during the study period will be included in the study. No patients will be enrolled as participants. Data gathered by the study will come from systematic observations in waiting rooms and routinely collected medical record data.

***Participant inclusion criteria:***

HIV/AIDS treatment facility inclusion:

The participating treatment facilities will have a combined total of at least 2,700 monthly patient visits during the study period. Each of the three HIV/AIDS treatment facilities meets the following inclusion criteria:

1. located in a jurisdiction with high AIDS prevalence;
2. managing more than 500 unique HIV-positive patients annually;
3. serving minority persons such that minimally 55% of the clinic population is African American and Hispanic/Latino;
4. at least 163 patients have unsuppressed viral load;
5. utilizing an electronic medical record (EMR) system; and
6. not participating in another behavioral intervention research project during the historical comparison data period (August 2015 through May 2016) or during the 10 month intervention period (June 2016 through March 2017)

Waiting room observation study inclusion:

1. Present in the waiting room during the 8 hours of observation at each HIV/AIDS treatment facility. To be included, observation subjects must also be situated in the waiting room in a location where they can be unobtrusively observed.
2. Since we are not interacting with observation subjects, we cannot determine their ages accurately (e.g., distinguish if a person is 17 versus 18 years old) or differentiate patients from friends who came with them. We will observe all persons in the waiting room, except for children who might be present in the company of adults.

HIV-positive patient medical record study inclusion:

Patients whose medical record data will be collected will be HIV-positive and 18 years of age or older. Data will be collected for both males and females. One inclusion criterion for participating HIV/AIDS treatment facilities is that the patient population must be at least 55% African American and/or Hispanic/Latino, so a minimum of 55% of subjects will be African-American or Hispanic/Latino.

***Participant exclusion criteria:***

HIV-positive patients who are under the age of 18 will be excluded from the patient medical record part of the study.

***Estimated number of participants*:**

Waiting room observational data: We estimate that the total number of patients who will be observed during the eight-hour observation period at the three treatment facilities will be between 192 and 336. Data collection for the observation will occur between June and July 2016.

HIV-positive patient medical record data: We estimate that the total number of patients over the three treatment facilities will range from 2,000-4,000, and the number of patient medical records over the 20-month investigation period will range from 8,000-16,000. Data abstraction from the medical records will occur from June 2016 through March 2017. These data include historical comparison medical data from August 1, 2015 through May 31, 2016 and intervention period medical data from June 1, 2016 through March 31, 2017.

***Sampling, including sample size and statistical power:***

The sample for the systematic observation will be a convenience sample of all patients who are present in the waiting room during the 1 day of observation at each facility. A maximum of 8 observational assessments will be done at each facility (32 total assessments). Each observational assessment will consist of systematic observation of all waiting room patients during one video cycle and systematic observation of four waiting room patients during the next video cycle. The four patients will be chosen randomly among patients who have a clear view of the video with the intent of obtaining diversity regarding age, gender, and race/ethnicity. See Appendix 2 for the observation protocol for additional details. The unit of analysis will be the individual patients. We do not expect to revise the sampling estimates for the study.

The sample for the patient medical record data will include all HIV-positive clinic patients and their records during the historical comparison period and the intervention period. Simplified sample size calculations were performed for rejecting the null hypothesis of equality in the proportions of successes for a specific outcome, assuming equal numbers of patients in each period. These calculations correspond to an analysis using Fisher’s exact test with a two-sided significance level of α = 5%.

For the primary outcome of medication adherence (achieving viral load suppression at 6 months), if the true proportions of successes for the two periods are 70% and 77% (historical comparison to intervention, 7% absolute improvement, 10% relative improvement), then sample sizes of 650 patients prescribed ART per period will be required to have 80% power to reject this null hypothesis. Allowing for an annual attrition rate of 25%, then sample sizes of 1157 patients per period will be required to have 80% power to reject this null hypothesis.

For the secondary outcome of retention in care at 3-7 months of follow-up, if the true proportions of successes for the two periods are 75% and 82% (historical comparison to intervention, 7% absolute improvement, 9% relative improvement), then sample sizes of 566 patients per period will be required to have 80% power to reject this null hypothesis. Allowing for an annual attrition rate of 25%, then sample sizes of 1008 patients per period will be required to have 80% power to reject this null hypothesis.

For the secondary outcome of treatment initiation (treatment-naïve patients receiving an ART prescription on the index visit), if the true proportions of successes for the two periods are 50% and 60% (historical comparison to intervention, 10% absolute improvement, 20% relative improvement), then sample sizes of 407 treatment-naïve patients per period will be required to have 80% power to reject this null hypothesis. (Attrition is not a factor in this calculation.)

This study is not powered to detect significant differences in partner protection (STD incidence) between the two study periods.

***Recruitment & Enrollment:***

Individual participants will not be recruited or enrolled.

HIV-positive patient medical record data:

No subjects will be recruited and enrolled. All adults who are HIV-positive patients in the three participating facilities during the study period will be included in the study. Subjects will be identified as HIV-positive patients through the facilities’ EMRs.

Waiting room observational data:

No subjects will be recruited and enrolled. All adults in the waiting room during the 8-hour observation period will be unobtrusively observed. Waiting rooms that are only for HIV-positive patients will be used (i.e., not mixed waiting rooms), so all adults in the waiting room will be assumed to be living with HIV and included in the observation. Patients will not be approached in any way by the observer, but will be unobtrusively observed to gauge their level of engagement with the video. To be included, observation subjects must also be situated in the waiting room in a location where they can be seen by the observer.

***C. VARIABLES/INTERVENTIONS***

***Variables:***

Patient medical data will be collected from the EMR, de-identified at the clinic using a computer-generated algorithm, and placed on an encrypted USB drive before mailing to the data manager for analysis. The following patient medical data will be collected:

- unique project ID number,
- gender,
- gender identity (if available),
- race/ethnicity,
- age,
- HIV risk factor,
- clinic visit dates,
- no-shows for scheduled outpatient visits,
- case management center visit dates (for Miami site only),
- date of HIV diagnosis,
- date of ART initiation,
- ART regimens,
- CD4 counts and test dates,
- viral loads and test dates,
- STD lab results and test dates

Observation assessments will include all patients in the waiting room, and more detailed observation of select patients.

Observation variables for the entire waiting room:

- Number of patients by apparent age
- Number of patients by apparent race/ethnicity
- Number of patients by apparent gender
- Number of patients who have a clear view of a video monitor
- Number of patients in the waiting room that the observer can see (to measure engagement with video)
- Number of patients in the waiting room who were present for the previous cycle
- General observations about engagement with each video segment for all patients for one cycle of the video

Observation variables for up to 4 select patients and up to 4 replacement patients:

- Apparent age of each patient
- Apparent race/ethnicity of each patient
- Apparent gender of each patient
- The duration of engagement with video for each video segment
- Reactions and watching behaviors of patient during each video segment
- General comments relative to engagement of the patients for one cycle of the video

***Study instruments, including questionnaires, laboratory instruments, and analytic tests:***

Patient medical data will be extracted from the facilities’ EMRs.

An online data collection instrument was developed to record data for the waiting room observations (see Appendix 3: Observation Instrument). This instrument consists of both qualitative and quantitative items, and is meant to record the demographic make-up of waiting room patients, and to observe them both generally and focusing on four patients at a time for more specific information with regard to their engagement with the video.

***Intervention or treatment:***

The intervention that will be tested will be a short “soap opera” style waiting room video with three interwoven vignettes that incorporate key prevention messages aimed at increasing early treatment initiation, treatment adherence, retention in care, and partner protection for people with HIV. The video will contain characters who represent various populations, including women, gay men, heterosexuals, African-Americans, and Latinos. The video will also include a short animation focusing on treatment adherence. The video is meant to play in the waiting room throughout the day for 10 months. Optimally, the video will play on a continuous loop. Some facilities may need short pauses between video replays based on their local circumstances. If this need arises, the pause interval will be discussed with and approved by CDC.

***Outcomes and minimum meaningful differences:***

The possible intervention outcomes during the intervention period compared to the 10 month period prior, include:

1. Increase in percent of patients who are retained in care (i.e., have clinic visit 3-7 months of follow-up)
2. Increase in percent of treatment-naïve patients receiving an ART prescription on the index visit
3. Increase in percent of patients achieving viral load suppression at 6 months (<50 RNA copies/mL plasma)
4. Decrease in incident sexually transmitted disease diagnoses among patients

The possible outcomes from the observation include the detection of certain video segments or parts of segments that are less engaging to viewers.

*Meaningful differences:*

As a practical matter, a 10% difference in patient medical outcomes is meaningful.

***Training for all study personnel:***

Observer will receive training before conducting the one day of unobtrusive systematic observation in the beta-test facility. This will include training on the observation protocol and use of the data collection instrument. Observer will be trained specifically to not approach any patients in the waiting room, not to make eye contact with patients, and to stop observing any patient that appears uncomfortable. EMR data staff will be trained on how to mail the data to the data manager on an encrypted USB drive that will be supplied to each facility. Both the PI and the data manager have completed human subjects protections training and are HIPAA certified.

***D. DATA HANDLING AND ANALYSIS***

***Data collection:***

Observational data: A detailed observation protocol is outlined in Appendix 2: Observation Protocol.

Patient medical data: De-identified patient medical data will be supplied by the facilities. The data will consist of data that each facility is already collecting in their EMR. The data will be de-identified by staff at each facility before being placed on an encrypted USB drive.

***Data entry, editing and management, including handling of data collection forms, different versions of data, and data storage and disposition:***

Observational data will be entered into Survey Monkey (a commercially available online survey solutions service) by the observer during the 8 hours of systematic observation at each beta-test facility. We will use the SSL encryption feature on Survey Monkey, which ensures the security of the data as it moves between the tablet computer and Survey Monkey servers, and during downloading of the data. Survey Monkey uses Verisign certificate Version 3, 128 bit encryption. The data will be stored in the Survey Monkey database until the end of the study. The observational data will not contain any information that could identify any of the patients in the waiting room (See Appendix 3: Observation Instrument). The Beta-Test Manager will be responsible for downloading the data from Survey Monkey in Excel format. He will clean the data, place it on an encrypted USB drive, and mail it to the CDC for analysis. The PI will destroy the observational dataset at the end of the project after CDC has confirmed receipt and approval of the data.

The patient medical record data will be de-identified at the HIV/AIDS treatment facilities by facility staff before data are transferred onto an encrypted USB drive. No Personally Identifiable Information (PII) will be included. The patient ID number will be changed into a random project ID number using an algorithm before the data are transferred to the USB drive. The data that are transferred to the encrypted USB drive will be checked for PII before and after placing it on the USB drive. After transferring the data onto the encrypted USB drive, the USB drive will be mailed or shipped by express mail using FedEx to the data manager. This transfer of data will occur at three points during the 10 month intervention period. Pre-paid mailers will be supplied to each participating facility for each transfer. When the data manager receives the data, she will immediately transfer it to a secure laptop that is not connected to the internet (the wireless card will be removed). This laptop will be stored in a locked cabinet in her office, which is also locked when the data manager is not inside. The laptop will be password protected, and the password will be changed every 60 days. SAS and SPSS data analysis software, including any updates, will be installed on this laptop using a CD/DVD only. The data manager will merge data from the three facilities into a cross-facility file and will run consistency and error checks. The central, cross-facility database will undergo a final cleaning to ensure that all inconsistencies and queries have been resolved before the final patient medical record dataset is submitted to the CDC. The data manager will destroy the final patient medical record datasets and back-ups at the end of the project after CDC has confirmed receipt and approval of the data.

Both the data manager and the treatment facility staff responsible for de-identifying and transferring the data will be required to sign a Rules of Behavior document which is required by the CDC (See Appendix 1: Rules of Behavior document). Outside of the treatment facilities, only the data manager will have access to the data before its transfer to the CDC. Sentient will forward all FedEx tracking e-mails to the Contact Officer Representative. CDC will monitor the movement of USB drives from facilities to the data manager and from the data manager to the CDC.

***Quality control/assurance:***

A pilot transfer of patient medical data will occur prior to collection of study data to work out any issues with the data transfer process. The data manager will run data consistency and error checks after receiving the data from each facility at the three points of patient medical data transfer. If there are issues with the data, these will be communicated to the PI, who will in turn inform the facility staff until the issue is rectified. The online systematic observation instrument will help ensure that observational data are collected consistently. Quality assurance for the observational data will include training of observer to follow the observation protocol, training the observerto use the online observational data collection instrument correctly, and practicing using the instrument. The Beta-Test Manager will monitor the data as it is collected, check for any issues, and re-train observer if any issues are found.

***Data analysis plan, including statistical methodology and planned tables and figures:***

Analysis of observational data: Patient engagement with the video during the first half of each assessment (i.e., all waiting room patients) will be analyzed qualitatively and separately for each of the six video segments (i.e., opening, three stories, and two animations). Codebooks for the content analyses will be developed after the data are received. Content analyses of the observations will look for number of patients who watch or ignore the segment and if the segment elicits any positive or negative responses. Relationships will be sought between frequency of disengagement (i.e., number of patients who ignored or exhibited a negative reaction during a segment) and characteristics of the disengaged patients (i.e., apparent age, gender, race/ethnicity). Patient engagement during the second half of each assessment (i.e., four select patients) will be analyzed the same way; however, analysis will attempt to identify within segments the scenes that are associated with patient disengagement. In addition, data from the second half of the assessments will be evaluated quantitatively for estimated duration of engagement with each segment. Patterns of disengagement across the three facilities will be sought. We will use EZ-Text qualitative software to assist with our qualitative data analysis.

Analysis of patient medical data: For analytical purposes index visits are defined as a patient’s first HIV care clinic visit during a beta-test period (i.e., the historical comparison period or the intervention period). Patients may have index visits in both time periods. The index visit in each period will be time zero. Initiation of ART will be defined as the proportion of treatment-naïve patients (i.e., no previous record of an ART prescription) who are prescribed ART (as per the current treatment guidelines) on their index visit. Medication adherence will be defined as the proportion of patients prescribed ART who achieve viral load suppression (<50 copies/mL) at least 40 days after their index visit. Retention in HIV medical care is defined as the proportion of patients who are retained in care at 3 to 7 months of follow-up from their index visit. Partner protection will be assessed using a proxy measure—incident STDs. Incident STD is defined as the proportion of patients who do not have an incident STD as a function of time since their index visit. Incident STD is defined as a microbiologically confirmed STD diagnoses noted in patients’ electronic medical records (EMRs). Kaplan-Meier methods will be used to estimate the proportion of patients who achieve intervention outcomes. The analyses may be adjusted for patient-level covariates, such as age, race, gender, baseline viral load suppression, and baseline STD, as well as the three beta-test treatment facilities.

***Information management and analysis software:***

The data manager will use SAS and SPSS to manage and clean the patient medical data and Excel to manage and clean the observational data. The data manager will mail the cleaned data to CDC on an encrypted USB drive. CDC will use SAS and SPSS to analyze the patient medical data and EZ-Text and Excel to analyze the observational data.

***Bias in data collection, measurement, and analysis:***

Biases with regard to the observation: Observation bias could affect the validity of the data. This bias will be minimized by having only one observer and training him to conduct the observation in a systematic fashion using an online instrument to guide the data collection, which will enhance observer consistency. Bias is also minimized by the data collection instrument requiring a low level of observer inference or interpretation of observed behaviors. In a waiting room patients are a collection of individuals and not participants in an interactive group, so the presence of a non-interacting observer should not influence patient behavior. Bias from expectancy effects will be offset by checking for correspondence between observed disengagement and medical outcomes addressed during segments when many patients were disengaged. There could be bias due to an inability of the observer to correctly recognize the HIV-positive patient characteristics (race/ethnicity, age, gender) or whether or not patients are truly engaged with the video or merely looking in the direction of the video screen. To a certain extent, this bias is inherent in the study design, and would be difficult to avoid without approaching the patients. Measurement bias could affect the generalizability of the data. Observations are limited to once per facility. Bias is minimized by conducting observations for eight hours, for a total of 24 assessments and an estimated 192 to 336 patients observed across all three facilities. The eight-hour observation period will minimize time-of-day bias but will be affected by day-of-week and month-of-year differences. Observations will be conducted during the first two months of the intervention period to minimize potential bias from previous exposure to the video.

Biases with regard to the patient medical data:

Bias is inherent in the study because the 10-month data collection timeframe is less than the 12 months required by core indicators for the outcomes being evaluated. Patient populations among the facilities are heterogeneous, so combining their medical data cannot be done reliably. Bias can occur from loss to follow-up (i.e., failure to return to the clinic) in either beta-test period. This attrition will be adjusted for analytically. Use of historical comparison will tend to exaggerate treatment effect as patients in this group benefit from amount of health care over time. Bias also can result from differences in individual medical care providers recording medical tests performed and treatment regimens prescribed and not performing medical tests at each clinic visit. Bias also can result from providers not testing all potentially infected anatomical sites for STDs. Bias may be introduced if there is a change in treatment guidelines during the 20-month data collection period.

***Intermediate reviews and analyses:***

Patient medical data will be collected from the facilities at three points over the 10 month study period. At each point, it will be transferred to the CDC for analysis. In addition, the PI will track the progress of the study and report this information to the CDC on a monthly basis, or more frequently.

***Limitations of study:***

This study is intended to provide specific data that will be used to refine the video intervention. These study results are not meant to be applicable to other interventions. A major limitation of the patient medical data part of the study is that it will not demonstrate conclusively that outcomes observed are due to exposure to the video. Available funding only allows 10 months of data collection, not 12 months as required by core indicators for the outcomes. Also there is no way to measure actual video exposure or dosage. With this study design, we can only observe associations between outcomes and presumed exposure. Limitations of the observational data are that observations will be conducted on only one day so data redundancy may not be reached, and the observer cannot be certain that the apparent demographics of the patients are 100% accurate or that patients are actually engaged with the video. Despite this, we believe the observations will allow for meaningful information that can be used to improve the video.

***E. HANDLING OF UNEXPECTED FINDINGS AND ADVERSE EVENTS***

***Response to new or unexpected findings and to changes in the study environment:***
New or unexpected findings will not be identified until data are analyzed at the end of the 10-month beta-test. All findings will inform the video refinement and improvement to increase its effectiveness among HIV-positive persons in medical care settings.

The PI will communicate by phone or email at least monthly with facility managers to deal with any changes which might arise in the study environment. Specifically, the PI will ensure that there are no issues with regard to playing the video or collection of patient data from the EMR. During the monthly communication, he will check that the video has been played each day and that the video is looping at a minimum of 20-minute intervals. Facility managers will be asked to report to the PI immediately if there are any issues, such as equipment malfunctioning or missing equipment. In the event of malfunctioning or missing equipment, the PI will work with the facility manager to replace the equipment. If there are changes in the EMR software used at the facilities, the PI and data manager will work with the facilities’ EMR data staff to make sure that all of the required variables continue to be collected.

***Identifying, managing, and reporting adverse events or incidents:***

Because the study involves using pre-existing, de-identified medical data and unobtrusive observation in public waiting rooms, we believe that the possibility of adverse events is minimal. However, if an adverse event occurs, such as the inadvertent placing of PII on the encrypted USB drive by the facility’s staff, this would be reported to CDC within 1 hour of discovery. The facility staff person responsible for placing the PII on the USB drive would be re-trained to de-identify and remove PII from the patient medical data.

***F. SHARING STUDY RESULTS***

***Anticipated products or inventions resulting from the study and their use:***

This study will result in a refined waiting room video for HIV/AIDS treatment facility waiting rooms that will be used as an intervention to improve HIV treatment adherence, retention in care, and sexual risk reduction for individuals with HIV. After refinement, the video will be available for use by HIV/AIDS treatment facilities across the US.

***Disseminating results to public:***

A data sharing plan will be developed explaining how and when CDC will make project data publically available. Publication guidelines and agreements will be developed as well.

**IV. HUMAN SUBJECTS PROTECTIONS**

***A. INFORMED CONSENT***

***Procedures for implementing and documenting informed consent:***

A waiver of written informed consent is being requested because the research presents no more than minimal risk to the subjects and because no personal identifying information will be collected. The patient medical data will be extracted from existing medical record data from the treatment facilities’ EMRs and de-identified by a computer algorithm. Furthermore, the systematic observations will take place unobtrusively in a public location.

***B. RISKS & BENEFITS***

***Description of risks (physical, social, psychological, economic, other) to the individual or group. Include methods to minimize risks:***

The proposed research presents no more than minimal risk to subjects. Some waiting room patients may feel uncomfortable if they notice that they are being observed. However, the trained observer will make every effort to be as unobtrusive as possible and will not approach any waiting room patients. In addition, there is slight risk that some PII could end up in the data on the encrypted USB drive. This is unlikely, however, as the medical data will be de-identified by treatment facility staff before it is transferred to the encrypted USB drive, and then checked once more after this transfer. The fact that the USB drive itself is encrypted adds another layer of protection. Upon receiving the USB drive, the data manager will immediately check the data for PII before loading the data onto the secure laptop. If any PII was present, the data manager would report this to the CDC within 1 hour of discovery.

***Description of anticipated benefits to the research participant*:**

The study will not enroll research participants. We anticipate that the beta-test version of the video will result in improved outcomes for patients in the waiting room when the video is shown with regard to HIV treatment adherence, retention in care, and sexual risk reduction. Consequently, this will lead to better health outcomes for patients with HIV and their sex partners.

***Description of the potential risks to anticipated benefit ratio:***

No participants will be enrolled in the study; however, patients will be exposed to the intervention video. Benefits of exposure to the video are increased likelihood of improved adherence to medication and retention in medical care and decreased likelihood of transmitting HIV to sex partners. This greatly outweighs the risk of becoming uncomfortable due to sensitive topics portrayed in the video. We believe that the overall risk classification for this study is minimal risk. As the final video will be made available for HIV/AIDS treatment facilities across the US, it has the potential for widespread improvement of the health of many people with HIV, as well as for their sex partners. We therefore believe that the potential risks of this investigation are greatly outweighed by the anticipated benefits.

***C. PRIVACY & CONFIDENTIALITY***

***Provisions for protecting privacy/confidentiality***

Patient medical data will be de-identified by treatment facility staff before it is transferred to the encrypted USB drive, and then checked once more after this transfer. The fact that the USB drive itself is encrypted adds another layer of protection. Upon receiving the USB drive, the data manager will immediately check the data for PII before loading the data onto the secure laptop. If any PII was present, the data manager would report this to the CDC within 1 hour of discovery. After the data manager confirms the data contain no PII, she will immediately transfer it to a secure laptop, which is not connected to the internet (the wireless card will be removed). This laptop will be stored in a locked cabinet in her office, which is also locked when the data manager is not inside. The laptop will be password protected, and the password will be changed every 60 days. The data manager and the facility staff responsible for de-identifying the data will be required to sign a Rules of Behavior document which is required by the CDC to protect against any identification of study participants. No identifying data will be published or released outside of the project.

***Statement about need or lack of need for Assurance or Certificate of Confidentiality:***

We do not anticipate that any formal confidentiality protections will be needed for this research, as we will not be enrolling any participants, and the study will consist of collecting de-identified patient medical data and unobtrusive observational data in public waiting rooms.

***Emergency care:***

As no patients will be enrolled in this study, there is no risk of an emergency as a result of this study. If an emergency occurred with a patient while they were in the waiting room, they would be in a clinic or HIV case-management office where staff are trained to deal with health emergencies.

***Description and justification of reimbursements or incentives that will be used:***

No participants will be enrolled; therefore, no reimbursements or incentives will be used.

***Notifying participants of their individual results:***

No participants will be enrolled; therefore, there will be no individual results.

***Notifying participants of study findings:***

No participants will be enrolled; therefore, there will be no one to be offered the option of receiving overall study findings.

**V. REFERENCES**

# Fisher J.D., [Smith L.R](http://www.ncbi.nlm.nih.gov/pubmed?term=Smith%20LR%5BAuthor%5D&cauthor=true&cauthor_uid=21406979)., [Lenz E.M](http://www.ncbi.nlm.nih.gov/pubmed?term=Lenz%20EM%5BAuthor%5D&cauthor=true&cauthor_uid=21406979). (2010). Secondary prevention of HIV in the United States: past, current, and future perspectives. *J Acquir Immune Defic Syndr*, 55, Suppl 2:S106-15.

2 National Institutes of Health. (2013). Guidelines for the Use of Antiretroviral Agents in HIV-1-Infected Adults and Adolescents. Retrieved from: http://aidsinfo.nih.gov/contentfiles/lvguidelines/adultandadolescentgl.pdf

3 Centers for Disease Control and Prevention. (2012). Fact Sheet: HIV in the United States: The Stages of Care. Retrieved from: http://www.cdc.gov/hiv/pdf/research_mmp_StagesofCare.pdf

^4^ Centers for Disease Control and Prevention. *Compendium of Evidence-Based HIV Behavioral Interventions: Medication Adherence Chapter*. Retrieved from: http://www.cdc.gov/hiv/topics/research/prs/ma-chapter.htm.

**VI. APPENDIX MATERIALS**

**APPENDIX 1: RULES OF BEHAVIOR DOCUMENT**

**APPENDIX 2: OBSERVATION PROTOCOL**

**APPENDIX 3: OBSERVATION INSTRUMENT**

**Appendix 1: Rules of Behavior Document**

Prevention with Positives-Antiretroviral Therapy (PWP-ART Video) Rules of Behavior (RoB)

All users collecting, storing or transferring PWP-ART Video study participant information must read these rules and sign the accompanying acknowledgement form. This acknowledgement must be signed annually to reaffirm knowledge of, and agreement to adhere to the PWP-ART Video RoB.

These rules cannot account for every possible situation. Therefore, personnel shall use their best judgment and highest ethical standards to guide their actions.

I assert my understanding that:

- Collection, storage, and transfer of Sensitive Information (SI) must comply with Federal Government, Department of Health and Human Services (HHS), and Centers for Disease Control and Prevention (CDC) policies and standards, and with applicable laws;
- SI for the purpose of the PWP-ART Video study includes patient demographic, HIV status, and laboratory test result information for study participants;
- Unauthorized access to information is prohibited, Authorized users shall not share or loan their credentials with any individual in order to allow that individual to gain access to the PWP-ART Video Laptop; and
- Users must prevent unauthorized disclosure of SI, Personal Health Information (PHI), or Personally Identifiable Information (PII) by following the procedures listed in the PWP-ART Video RoB.

For individuals with electronic access to the PWP-ART Video Laptop:

I shall:

- Only connect the PWP-ART Video Laptop to the internet once to initially install the system and analysis software required for the Laptop, after which no further connection to the internet will be allowed without written permission from the NCHHSTP ISSO Ralph Vaughn and the wireless card will be removed from the Laptop to prevent inadvertent connection to the internet;
- Update software on the PWP-ART Laptop that is necessary to perform job functions by using software disks purchased off-the-shelf; If such disks are not available, obtain authorization from CDC NCHHSTP ISSO, Ralph Vaughn, before downloading software from the internet;
- Log-off or lock the PWP-ART Video Laptop and lock the office door when leaving it unattended;
- Store the CDC issued encrypted USB devices in a locked drawer or cabinet when not in use to prevent loss;
- Use a separate Administrative and User password for access to the Administrative functions of the Laptop and regular user functions.
- Create a password that complies with the following minimum requirements:
  - Minimum of 8 characters
  - Use at least one each of 3 of the 4 types of characters, Upper Case Letter, Lower Case Letter, Number, and Special Character
  - Change Administrative and User passwords at least every 60 days and set 2-week reminders on the Laptop;
  - Send report verifying password changes to the CDC Project Officer (Contracting Officer’s Representative)
- Use Federal Information Processing Standards (FIPS) 140-2 full disk encryption on the Laptop such as Symantec Drive Encryption (PGP Whole Disk Encryption)
- Use only CDC issued encrypted USB devices to transfer data to or from the Laptop and for back-up of data on the Laptop;
- Before analyzing any data from the encrypted USB device, check the data on the PWP-ART Video Laptop to ensure that there is no PII. If there is PII present, report this to the CDC Project Officer as described below.
- If PII is present, delete the PII before analyzing data.
- Complete HHS’s Security Awareness Training module before processing data and on an annual basis thereafter, as required by CDC policies.
  Link (<http://www.hhs.gov/ocio/securityprivacy/awarenesstraining/awarenesstraining.html> )
- Keep SI data out of sight when visitors are present;
- Only access SI necessary to perform job functions (i.e., need to know);
- Protect HHS information assets (HHS assets include but are not limited to hardware, software, and federal records) from unauthorized access, use, modification, destruction, theft, or disclosure and shall treat such assets in accordance with any information handling policies;
- Within 1 hour of discovery of a known or suspected security breach (lost or stolen SI; known or suspected security incidents; known or suspected information security policy violations or compromises; or suspicious activity), report to the CDC Project Officer (Contracting Officer’s Representative) who will then report the incident to the NCHHSTP ISSO. Known or suspected security incidents involve the actual or potential loss of control or compromise, whether intentional or unintentional, of SI maintained or in possession of contractors and third parties on behalf of CDC.

I shall not:

- Circumvent security safeguards including violating security policies or procedures or reconfigure systems except as authorized (i.e., violation of least privilege);
- Use another person’s account, identity, or password;
- Exceed authorized access to Share or disclose SI except as authorized;
- Store SI in public folders or other insecure physical or electronic storage locations;
- Transport, transmit or download SI unless such action is explicitly permitted by the manager or owner of such information and appropriate safeguards are in place per CDC policies concerning SI;
- Use SI for anything other than the purpose for which it has been authorized;
- Use sensitive CDC data for private gain or to misrepresent myself or CDC or any other unauthorized purpose;
- Transfer SI to **personal** mobile devices such as laptops, universal serial bus (USB) drives, or on remote/home systems;
- Knowingly or willingly conceal, remove, mutilate, obliterate, falsify, or destroy information for personal use by myself or others;
- Connect the PWP-ART Video Laptop to a server, network, or the internet either through direct or remote connection (i.e., wireless, modem);
- Load unapproved software from unauthorized sources on the PWP-ART Video Laptop;
- Use the PWP-ART Video Laptop without the following protections engaged before accessing sensitive PWP-ART Video study information:
  - Antivirus software with the latest updates;
  - A time-out function that requires re-authentication after no more than 30 minutes of inactivity; and
  - Approved encryption to protect SI stored on the PWP-ART Laptop and CDC issued USB drives.

The following are prohibited actions on the PWP-ART Video Laptop:

- Unethical or illegal conduct;
- Utilizing peer-to-peer software;

Rules for SI Applicable to All Users

Storage of SI

All SI should be stored within a CDC issued encrypted USB device or on the encrypted PWP-ART Video Laptop and stored in an area with limited access (behind a locked door, drawer, or cabinet), an access controlled electronic environment (keycard access), or under the physical control of an authorized individual. SI will not be stored on paper.

Backup of SI

All PWP-ART Video study data stored on the laptop will be backed up weekly to a separate CDC issued encrypted USB device only. Weekly backups will be maintained for at least two weeks, after which they can be deleted from the USB device. The Backup USB device will be larger than 4GB and will be coordinated with the program for delivery and issue.

Transferring SI

SI may be transferred from the beta-test clinics to CDC issued encrypted USB device and mailed to Dr. Marjan Javanbakht only. SI will not be emailed or otherwise electronically transmitted to or from any source to the PWP-ART Video Laptop.

SI Disposal and Destruction Requirements and Methods

When no longer required, SI recorded on the CDC issued encrypted USB or PWP-ART Video Laptop will be destroyed by using an approved electronic wiping software, such as BC Wipe, degaussing or physically destroyed beyond use.

**Individual Acknowledgement Statement**

I have read the *PWP-ART Video Rules of Behavior* (RoB), and understand and agree to comply with its provisions. I understand that exceptions to the PWP-ART Video RoB must be authorized in advance in writing by the NCHHSTP ISSO or his/her designee. I also understand that violation of laws, such as the Privacy Act of 1974, copyright law, and 18 USC 2071, Chapter 47, which the PWP-ART Video RoB draws upon, can result in monetary fines and/or criminal charges that may result in imprisonment.

______________________________ ________________

Signature Date

_______________________________ ________________

Printed Name Agency

Original to be retained by the PWP-ART Video Project Coordinator

**Appendix 2: Observation Protocol**

**Waiting Room Observation Protocol**

Scheduling: Systematic observations will be conducted within the first 3 months of the beta-test period to increase the likelihood that the patients being observed are seeing the video for the first time.

Checking in: At the beginning of the shift, observer will check in with the clinic manager, determine which video playback option is being used (i.e., continuous play, 10-minute interval between cycles, 20-minute interval), determine which waiting room seats do **not** offer a clear (unobstructed) view of a video monitor, and find a seat to begin the observation.

Finding a spot for the observation: Observer will find an open seat where he/she will be able to watch patients’ reactions from the most unobtrusive location possible. The patients should not be behind the observer. The observer may change positions if his/her position is not working well.

All data will be entered on a tablet using a Survey Monkey data collection instrument. Each assessment will have four sections:

1. **Initial waiting room conditions**
2. **Systematic observation of all waiting room patients during one video cycle**
3. **Systematic observation of select waiting room patients during the next video cycle**
4. **Summary comments**

The observer will complete one assessment for every two cycles of the video. The observer will continue to complete the assessments until he/she has completed an 8-hour shift (6-8 assessments).

**1. Initial waiting room conditions**

Survey Monkey will stamp the date and time automatically. The other variables collected in this section include:

- Clinic where observations are being done
- Video version (English only/ English with Spanish Subtitles)
- Audibility (i.e. can video be heard throughout the waiting room)
- Number of monitors playing video in waiting room
- General conditions of waiting room that would impact patients’ engagement with video

**2. Systematic observation of waiting room patients**

Observer will begin this observation at the beginning of the video (i.e. video Opening shots and title) and will continue this observation through each segment of stories or animation in the video for one full cycle of the video. The observer will concentrate on patients who were not present during the previous video cycle. The observer will look around the room going from patient to patient, covering all patients that can be seen from his/her vantage point, to observe their reactions. The main objective of this section is to note any points in the video that seemed to hold patients’ attention, not hold attention, or elicit a positive or negative reaction such as laughter, discussion, or disengagement with the video. Patients who are multi-tasking while watching the video will be considered to be watching the video. Observer will use the time during the credits to note additional general observations for this cycle.

The variables collected in this section include:

- Number of patients by apparent age
- Number of patients by apparent race/ethnicity
- Number of patients by apparent gender
- Number of patients who have a clear view of a video monitor
- Number of patients in the waiting room that the observer can see (to measure engagement with video)
- Number of patients in the waiting room who were present for the previous cycle.
- General observations about engagement with all video segments for all patients for this cycle

**3. Systematic observation of select waiting room patients**

Observer will randomly choose four patients who have a clear view of the video, attempting to get a diverse sample regarding age, gender, and race/ethnicity. Patients who were not present in the previous cycle should be chosen, unless there are fewer than four patients in the waiting room who were not present during the previous cycle. If one or more of the four patients was present for the previous cycle, note this on the Survey Monkey instrument.

The observer will watch these four patients for one video cycle. Observer will begin this observation at the beginning of the video (i.e., Opening) and will continue this observation through each segment of stories or animation in the video for one full cycle of the video. The observer will record all reactions and behaviors that occur during each segment. For example, a patient may begin watching the video, have a negative reaction, and stop watching the video during a segment. If a patient is absent during one or more segments of the video during the cycle, the observer will check the “N/P” box for that patient’s reactions for those segments (e.g., if the patient leaves in the middle of a story, the observer will mark what occurred for that patient up until the time he or she leaves, and enter N/P for the rest of his or her segments). If there are fewer than 4 people in the waiting room to observe initially, the observer will enter N/P for the missing patients’ segments. However, if one or more patients subsequently enter the waiting room during this cycle, the observer should begin recording these patients.

Replacements: If one or more replacements are needed, the observer will try to match the replacement demographics in terms of gender, race/ethnicity, and age (in that order). The observer should try to identify potential replacements at the beginning of the cycle in case they are needed. If any of the initial 4 patients being observed leaves, the observer will begin observing a replacement patient (and will add the replacement’s demographics to the instrument). The replacements will be numbered in the order in which they occur from 1 to 4. The observer should take care to remember which patient is which (e.g. choose #1 and go clockwise to #4). If one of the original patients comes back, the observer will stop watching the replacement at that point and go back to watching the original patient. The observer will mark N/P for any segments for which either a replacement or an original patient are not present.

The observer will use the time during the credits to note additional general observations for the cycle. The main point of this section is to note *in detail* any points in the video that seemed to hold attention, not hold attention, or elicit a particular reaction such as laughter, discussion, or disengagement with the video specifically for the patients observed.

If any patient notices appears uncomfortable, the observer should not choose them as an observation subject or should stop observing them immediately (and begin observing a replacement) if the observation has already begun. If any patients appear to be uncomfortable, the observer should record the event in summary comments.

The variables collected in this section include:

- Apparent age of each patient
- Apparent race/ethnicity of each patient
- Apparent gender of each patient
- The duration of engagement with video for each video segment
- Reactions and watching behaviors of patient during each video segment
- Information relative to engagement of the patients for this cycle

**IV. Summary comments**

After having watched the video for two cycles (once for the entire waiting room and once for the 4 patients), the observer will note any additional or summary comments with regard to engagement with the video, including whether engagement appeared to vary by demographics and by stories or segment of stories. The observer also will note whether there was a significant difference in the general conditions of the waiting room conditions for the two cycles that could have impacted patients’ engagement with the video.

**Appendix 3: Observation Instrument**

# Waiting Room Video Observation Data Collection Instrument

*[Simulated Survey Monkey display]*

## 1. Initial Waiting Room Conditions

### 1a.In which clinic are you observing today?

| Clinic |  | ⯆ |
| --- | --- | --- |
|  | Clinic 1 |  |
|  | Clinic 2 |  |
|  | Clinic 3 |  |
|  | Clinic 4 |  |

### 1b. What version of the video is playing?

| Video Version |  | ⯆ |
| --- | --- | --- |
|  | English only |  |
|  | English with Spanish subtitles |  |

### 1c. Is the video audible in most areas of the waiting room?

| 🞅 | Yes |
| --- | --- |
| 🞅 | No |

### 1d. How many monitors are playing?

| Number of monitors |  | ⯆ |
| --- | --- | --- |
|  | 0 |  |
|  | 1 |  |
|  | 2 |  |
|  | 3 |  |
|  | More than 3 |  |

### 1e. Use the space below to note any issues with the video or conditions in the waiting room that would likely impact patients’ experience watching the video.

|  | ▲ |
| --- | --- |
|  |  |
|  | ⯆ |

| **Next** |
| --- |

## 2. Observation of All Waiting Room Patients

### 2a. How many of the patients in the waiting room have an unobstructed view of the video?

|  |
| --- |

### 2b. How many of the patients can you see clearly enough to observe their reactions to the video?

|  |
| --- |

### 2c. Number of patients by apparent race/ethnicity in the waiting room

| American Indian/Alaska Native |  |
| --- | --- |
|  |  |
| Asian |  |
|  |  |
| Black/African American |  |
|  |  |
| Hispanic/Latino |  |
|  |  |
| Native Hawaiian/Other Pacific Islander |  |
|  |  |
| White |  |
|  |  |
| Other/Unsure |  |

### 2d. Number of patients by apparent age in the waiting room

| 25 or younger |  |
| --- | --- |
|  |  |
| 26 or older |  |
|  |  |
| Unsure |  |

### 2e. Number of patients by apparent gender in the waiting room

| Male |  |
| --- | --- |
|  |  |
| Female |  |
|  |  |
| Unsure |  |

### 2f. How many of these patients were present for any portion of the previous video cycle?

|  |
| --- |

### 2g. Opening: Enter general comments and any information relevant to patient engagement that you observed during this segment. For example, at what part of the opening did patients begin to take notice of the video? Did this seem to vary by race/ethnicity, age, or gender?

|  | ▲ |
| --- | --- |
|  |  |
|  | ⯆ |

### 2h. Story 1: Enter general comments and any information relevant to patient engagement that you observed during this segment. For example, did one part of the story seem to be particularly engaging or not engaging? Did this seem to vary by race/ethnicity, age, or gender?

|  | ▲ |
| --- | --- |
|  |  |
|  | ⯆ |

### 2i. Animation 1: Enter general comments and any information relevant to patient engagement that you observed during this segment. For example, did one part of the animation seem to be particularly engaging or not engaging? Did this seem to vary by race/ethnicity, age, or gender?

|  | ▲ |
| --- | --- |
|  |  |
|  | ⯆ |

### 2j. Story 2: Enter general comments and any information relevant to patient engagement that you observed during this segment. For example, did one part of the story seem to be particularly engaging or not engaging? Did this seem to vary by race/ethnicity, age, or gender?

|  | ▲ |
| --- | --- |
|  |  |
|  | ⯆ |

### 2k. Animation 2: Enter general comments and any information relevant to patient engagement that you observed during this segment. For example, did one part of the animation seem to be particularly engaging or not engaging? Did this seem to vary by race/ethnicity, age, or gender?

|  | ▲ |
| --- | --- |
|  |  |
|  | ⯆ |

### 2l. Story 3: Enter general comments and any information relevant to patient engagement that you observed during this segment. For example, did one part of the story seem to be particularly engaging or not engaging? Did this seem to vary by race/ethnicity, age, or gender?

|  | ▲ |
| --- | --- |
|  |  |
|  | ⯆ |

| **Prev** |  | **Next** |
| --- | --- | --- |

## 3. Observation of Select Waiting Room Patients

### 3a. Patient Apparent Demographics

Age Race/ethnicity Gender Present for previous cycle

| Patient 1 |  | ⯆ |  |  | ⯆ |  |  | ⯆ |  |  | ⯆ |  |
| --- | --- | --- | --- | --- | --- | --- | --- | --- | --- | --- | --- | --- |
| Patient 2 |  | ⯆ |  |  | ⯆ |  |  | ⯆ |  |  | ⯆ |  |
| Patient 3 |  | ⯆ |  |  | ⯆ |  |  | ⯆ |  |  | ⯆ |  |
| Patient 4 |  | ⯆ |  |  | ⯆ |  |  | ⯆ |  |  | ⯆ |  |
| Replacement 1 |  | ⯆ |  |  | ⯆ |  |  | ⯆ |  |  | ⯆ |  |
| Replacement 2 |  | ⯆ |  |  | ⯆ |  |  | ⯆ |  |  | ⯆ |  |
| Replacement 3 |  | ⯆ |  |  | ⯆ |  |  | ⯆ |  |  | ⯆ |  |
| Replacement 4 |  | ⯆ |  |  | ⯆ |  |  | ⯆ |  |  | ⯆ |  |
|  | 25 or younger |  |  | American Indian/Alaska Native |  |  | Female |  |  | Yes |  |  |
|  | 26 or older |  |  | Asian |  |  | Male |  |  | No |  |  |
|  | Unsure |  |  | Black/African American |  |  | Unsure |  |  |  |  |  |
|  |  |  |  | Hispanic/Latino |  |  |  |  |  |  |  |  |
|  |  |  |  | Native Hawaiian/Other Pacific Islander |  |  |  |  |  |  |  |  |
|  |  |  |  | White |  |  |  |  |  |  |  |  |
|  |  |  |  | Other/Unsure |  |  |  |  |  |  |  |  |

### 3b. Opening: Mark reactions or behaviors for each patient you are observing. Mark N/P if patient was not present for this segment. (Note all that apply)

|  | Began watching | Positive reaction | Negative reaction | Stopped watching | N/P |
| --- | --- | --- | --- | --- | --- |
| Patient 1 | □ | □ | □ | □ | □ |
| Patient 2 | □ | □ | □ | □ | □ |
| Patient 3 | □ | □ | □ | □ | □ |
| Patient 4 | □ | □ | □ | □ | □ |
| Replacement 1 | □ | □ | □ | □ | □ |
| Replacement 2 | □ | □ | □ | □ | □ |
| Replacement 3 | □ | □ | □ | □ | □ |
| Replacement 4 | □ | □ | □ | □ | □ |

### 3c. Opening: Note the duration of engagement (i.e. amount of the opening watched) for each patient during this video segment. Mark N/P if a patient was not present for this segment.

|  | All of it | More than half | Less than half | None of it | N/P |
| --- | --- | --- | --- | --- | --- |
| Patient 1 | 🞅 | 🞅 | 🞅 | 🞅 | 🞅 |
| Patient 2 | 🞅 | 🞅 | 🞅 | 🞅 | 🞅 |
| Patient 3 | 🞅 | 🞅 | 🞅 | 🞅 | 🞅 |
| Patient 4 | 🞅 | 🞅 | 🞅 | 🞅 | 🞅 |
| Replacement 1 | 🞅 | 🞅 | 🞅 | 🞅 | 🞅 |
| Replacement 2 | 🞅 | 🞅 | 🞅 | 🞅 | 🞅 |
| Replacement 3 | 🞅 | 🞅 | 🞅 | 🞅 | 🞅 |
| Replacement 4 | 🞅 | 🞅 | 🞅 | 🞅 | 🞅 |

### 3d. Enter information relevant to patient engagement that you observed during the Opening. For example, did one part of the opening seem to hold attention, lose attention, or elicit laughter or comment?

|  | ▲ |
| --- | --- |
|  |  |
|  | ⯆ |

### 3e. Story 1: Mark reactions or behaviors for each patient you are observing. Mark N/P if patient was not present for this segment. (Note all that apply)

|  | Began watching | Positive reaction | Negative reaction | Stopped watching | N/P |
| --- | --- | --- | --- | --- | --- |
| Patient 1 | □ | □ | □ | □ | □ |
| Patient 2 | □ | □ | □ | □ | □ |
| Patient 3 | □ | □ | □ | □ | □ |
| Patient 4 | □ | □ | □ | □ | □ |
| Replacement 1 | □ | □ | □ | □ | □ |
| Replacement 2 | □ | □ | □ | □ | □ |
| Replacement 3 | □ | □ | □ | □ | □ |
| Replacement 4 | □ | □ | □ | □ | □ |

### 3f. Story 1: Note the duration of engagement (i.e. watching video) for each patient during this video segment. Mark N/P if a patient was not present for this segment.

|  | All of it | More than half | Less than half | None of it | N/P |
| --- | --- | --- | --- | --- | --- |
| Patient 1 | 🞅 | 🞅 | 🞅 | 🞅 | 🞅 |
| Patient 2 | 🞅 | 🞅 | 🞅 | 🞅 | 🞅 |
| Patient 3 | 🞅 | 🞅 | 🞅 | 🞅 | 🞅 |
| Patient 4 | 🞅 | 🞅 | 🞅 | 🞅 | 🞅 |
| Replacement 1 | 🞅 | 🞅 | 🞅 | 🞅 | 🞅 |
| Replacement 2 | 🞅 | 🞅 | 🞅 | 🞅 | 🞅 |
| Replacement 3 | 🞅 | 🞅 | 🞅 | 🞅 | 🞅 |
| Replacement 4 | 🞅 | 🞅 | 🞅 | 🞅 | 🞅 |

### 3g. Enter information relevant to patient engagement that you observed during Story 1. For example, did one part of the story seem to hold attention, lose attention, or elicit laughter or comment?

|  | ▲ |
| --- | --- |
|  |  |
|  | ⯆ |

### 3h. Animation 1: Mark reactions or behaviors for each patient you are observing. Mark N/P if patient was not present for this segment. (Note all that apply.)

|  | Began watching | Positive reaction | Negative reaction | Stopped watching | N/P |
| --- | --- | --- | --- | --- | --- |
| Patient 1 | □ | □ | □ | □ | □ |
| Patient 2 | □ | □ | □ | □ | □ |
| Patient 3 | □ | □ | □ | □ | □ |
| Patient 4 | □ | □ | □ | □ | □ |
| Replacement 1 | □ | □ | □ | □ | □ |
| Replacement 2 | □ | □ | □ | □ | □ |
| Replacement 3 | □ | □ | □ | □ | □ |
| Replacement 4 | □ | □ | □ | □ | □ |

### 3i. Animation 1: Note the duration of engagement (i.e. watching video) for each patient during this video segment. Mark N/P if a patient was not present for this segment.

|  | All of it | More than half | Less than half | None of it | N/P |
| --- | --- | --- | --- | --- | --- |
| Patient 1 | 🞅 | 🞅 | 🞅 | 🞅 | 🞅 |
| Patient 2 | 🞅 | 🞅 | 🞅 | 🞅 | 🞅 |
| Patient 3 | 🞅 | 🞅 | 🞅 | 🞅 | 🞅 |
| Patient 4 | 🞅 | 🞅 | 🞅 | 🞅 | 🞅 |
| Replacement 1 | 🞅 | 🞅 | 🞅 | 🞅 | 🞅 |
| Replacement 2 | 🞅 | 🞅 | 🞅 | 🞅 | 🞅 |
| Replacement 3 | 🞅 | 🞅 | 🞅 | 🞅 | 🞅 |
| Replacement 4 | 🞅 | 🞅 | 🞅 | 🞅 | 🞅 |

### 3j. Enter information relevant to patient engagement that you observed during Animation 1. For example, did one part of the animation seem to hold attention, lose attention, or elicit laughter or comment?

|  | ▲ |
| --- | --- |
|  |  |
|  | ⯆ |

### 3k. Story 2: Mark reactions or behaviors for each patient you are observing. Mark N/P if patient was not present for this segment. (Note all that apply.)

|  | Began watching | Positive reaction | Negative reaction | Stopped watching | N/P |
| --- | --- | --- | --- | --- | --- |
| Patient 1 | □ | □ | □ | □ | □ |
| Patient 2 | □ | □ | □ | □ | □ |
| Patient 3 | □ | □ | □ | □ | □ |
| Patient 4 | □ | □ | □ | □ | □ |
| Replacement 1 | □ | □ | □ | □ | □ |
| Replacement 2 | □ | □ | □ | □ | □ |
| Replacement 3 | □ | □ | □ | □ | □ |
| Replacement 4 | □ | □ | □ | □ | □ |

### 3l. Story 2: Note the duration of engagement (i.e. watching video) for each patient during this video segment. Mark N/P if a patient was not present for this segment.

|  | All of it | More than half | Less than half | None of it | N/P |
| --- | --- | --- | --- | --- | --- |
| Patient 1 | 🞅 | 🞅 | 🞅 | 🞅 | 🞅 |
| Patient 2 | 🞅 | 🞅 | 🞅 | 🞅 | 🞅 |
| Patient 3 | 🞅 | 🞅 | 🞅 | 🞅 | 🞅 |
| Patient 4 | 🞅 | 🞅 | 🞅 | 🞅 | 🞅 |
| Replacement 1 | 🞅 | 🞅 | 🞅 | 🞅 | 🞅 |
| Replacement 2 | 🞅 | 🞅 | 🞅 | 🞅 | 🞅 |
| Replacement 3 | 🞅 | 🞅 | 🞅 | 🞅 | 🞅 |
| Replacement 4 | 🞅 | 🞅 | 🞅 | 🞅 | 🞅 |

3m. Enter information relevant to patient engagement that you observed during Story 2. For example, did one part of the story seem to hold attention, lose attention, or elicit laughter or comment?

|  | ▲ |
| --- | --- |
|  |  |
|  | ⯆ |

### 3n. Animation 2: Mark reactions or behaviors for each patient you are observing. Mark N/P if patient was not present for this segment. (Note all that apply.)

|  | Began watching | Positive reaction | Negative reaction | Stopped watching | N/P |
| --- | --- | --- | --- | --- | --- |
| Patient 1 | □ | □ | □ | □ | □ |
| Patient 2 | □ | □ | □ | □ | □ |
| Patient 3 | □ | □ | □ | □ | □ |
| Patient 4 | □ | □ | □ | □ | □ |
| Replacement 1 | □ | □ | □ | □ | □ |
| Replacement 2 | □ | □ | □ | □ | □ |
| Replacement 3 | □ | □ | □ | □ | □ |
| Replacement 4 | □ | □ | □ | □ | □ |

### 3o. Animation 2: Note the duration of engagement (i.e. watching video) for each patient during this video segment. Mark N/P if a patient was not present for this segment.

|  | All of it | More than half | Less than half | None of it | N/P |
| --- | --- | --- | --- | --- | --- |
| Patient 1 | 🞅 | 🞅 | 🞅 | 🞅 | 🞅 |
| Patient 2 | 🞅 | 🞅 | 🞅 | 🞅 | 🞅 |
| Patient 3 | 🞅 | 🞅 | 🞅 | 🞅 | 🞅 |
| Patient 4 | 🞅 | 🞅 | 🞅 | 🞅 | 🞅 |
| Replacement 1 | 🞅 | 🞅 | 🞅 | 🞅 | 🞅 |
| Replacement 2 | 🞅 | 🞅 | 🞅 | 🞅 | 🞅 |
| Replacement 3 | 🞅 | 🞅 | 🞅 | 🞅 | 🞅 |
| Replacement 4 | 🞅 | 🞅 | 🞅 | 🞅 | 🞅 |

3p. Enter information relevant to patient engagement that you observed during Animation 2. For example, did one part of the animation seem to hold attention, lose attention, or elicit laughter or comment?

|  | ▲ |
| --- | --- |
|  |  |
|  | ⯆ |

### 3q. Story 3: Mark reactions or behaviors for each patient you are observing. Mark N/P if patient was not present for this segment. (Note all that apply.)

|  | Began watching | Positive reaction | Negative reaction | Stopped watching | N/P |
| --- | --- | --- | --- | --- | --- |
| Patient 1 | □ | □ | □ | □ | □ |
| Patient 2 | □ | □ | □ | □ | □ |
| Patient 3 | □ | □ | □ | □ | □ |
| Patient 4 | □ | □ | □ | □ | □ |
| Replacement 1 | □ | □ | □ | □ | □ |
| Replacement 2 | □ | □ | □ | □ | □ |
| Replacement 3 | □ | □ | □ | □ | □ |
| Replacement 4 | □ | □ | □ | □ | □ |

### 3r. Story 3: Note the duration of engagement (i.e. watching video) for each patient during this video segment. Mark N/P if a patient was not present for this segment.

|  | All of it | More than half | Less than half | None of it | N/P |
| --- | --- | --- | --- | --- | --- |
| Patient 1 | 🞅 | 🞅 | 🞅 | 🞅 | 🞅 |
| Patient 2 | 🞅 | 🞅 | 🞅 | 🞅 | 🞅 |
| Patient 3 | 🞅 | 🞅 | 🞅 | 🞅 | 🞅 |
| Patient 4 | 🞅 | 🞅 | 🞅 | 🞅 | 🞅 |
| Replacement 1 | 🞅 | 🞅 | 🞅 | 🞅 | 🞅 |
| Replacement 2 | 🞅 | 🞅 | 🞅 | 🞅 | 🞅 |
| Replacement 3 | 🞅 | 🞅 | 🞅 | 🞅 | 🞅 |
| Replacement 4 | 🞅 | 🞅 | 🞅 | 🞅 | 🞅 |

### 3s. Enter any information relevant to patient engagement that you observed during Story 3. For example, did one part of the story seem to hold attention, lose attention, or elicit laughter or comment?

|  | ▲ |
| --- | --- |
|  |  |
|  | ⯆ |

| **Prev** |  | **Next** |
| --- | --- | --- |

## 4. General comments

### 4a. Having watched the video for two cycles, what are your impressions regarding engagement with the video? Were there any stories, scenes, or parts of scenes that seemed to hold patients’ attention especially well? Were there any stories, scenes, or parts of scenes that did not seem to hold patients’ attention? Did anything change in the waiting room that could have affected engagement?

|  | ▲ |
| --- | --- |
|  |  |
|  | ⯆ |

| **Prev** |  | **Done** |
| --- | --- | --- |
