# Supplementary material for: Effects of a brief video intervention on treatment initiation and adherence among patients attending human immunodeficiency virus treatment clinics
Source: PLoS One. 2018 Oct 5;13(10):e0204599. doi: 10.1371/journal.pone.0204599 (PMC6173379; doi:10.1371/journal.pone.0204599)
Supplement: S3 Appendix — (PDF) [file pone.0204599.s003.pdf]

AIDS ACTION COALITION | thrive ALABAMA

July 19, 2018

Dear *Taking Care of Me* Project Team

We at the Thrive Clinic very much enjoyed working with you on the research and evaluation of the *Taking Care of Me* video-based intervention. We agree that the influx of new patients during the study, an increase of 23.5%, likely had a major impact on your observed findings. We acknowledge that the study used a different method other than the standard HRSA/HAB performance measures to measure ART medication adherence, viral load suppression, and retentions in care.

Using the HAB performance measures, Thrive reported an increase in retention in care (91.6% to 92%); an increase in the prescription of ART (94.2% to 96%); and a slight decrease in viral load suppression (84% to 81.2%) during the intervention period from June 2016 to March 2017. These measures are all above the 2020 targets detailed in the National HIV/AIDS Strategy.

At the same time, we acknowledge that your explanation for reductions in viral load suppression are reasonable using your methods, and the fact that new patients may not have had time to reach viral load suppression given newly started ART regiments during the 10-month follow-up period.

Thanks again for allowing Thrive to be a part of the study, and we look forward to future opportunities.

Sincerely

Pam DeLozier

600 St. Clair Avenue, Building 3, Huntsville, AL 35801 • (256) 536-4700 • *www.thrivealabama.org*
